# Prevalence and predictors of vector-borne pathogens in Dutch roe deer

**DOI:** 10.1186/s13071-022-05195-w

**Published:** 2022-03-05

**Authors:** Sara R. Wijburg, Manoj Fonville, Arnout de Bruin, Piet A. van Rijn, Margriet G. E. Montizaan, Jan van den Broek, Hein Sprong, Jolianne M. Rijks

**Affiliations:** 1 Dutch Wildlife Health Centre, Department of Biomolecular Health Sciences, Faculty of Veterinary Medicine, Utrecht University, Utrecht, The Netherlands; 2 Centre for Infectious Disease Control, National Institute for Public Health and the Environment, Bilthoven, The Netherlands; 3 Department of Virology, Wageningen Bioveterinary Research, Wageningen University and Research, Lelystad, The Netherlands; 4 Centre for Human Metabolomics, Department of Biochemistry, North-West University, Potchefstroom, South Africa; 5 Department of Population Health Sciences, Faculty of Veterinary Medicine, Utrecht University, Utrecht, The Netherlands

**Keywords:** *Capreolus capreolus*, *Anaplasma phagocytophilum*, *Babesia*, *Bartonella*, *Borrelia*, *Rickettsia*, *Neoehrlichia mikurensis*, Epizootic haemorrhagic disease virus, Bluetongue virus, Co-infection

## Abstract

**Background:**

The main objective of this study was to determine the prevalence of nine vector-borne pathogens or pathogen genera in roe deer (*Capreolus capreolus*) in the Netherlands, and to identify which host variables predict vector-borne pathogen presence in roe deer. The host variables examined were the four host factors ‘age category’, ‘sex’, ‘nutritional condition’ and ‘health status’, as well as ‘roe deer density’.

**Methods:**

From December 2009 to September 2010, blood samples of 461 roe deer were collected and analysed by polymerase chain reaction (PCR) for the presence of genetic material from *Anaplasma phagocytophilum*, *Bartonella* spp., *Babesia* spp., *Borrelia burgdorferi* sensu lato (s.l.), *Borrelia miyamotoi*, *Neoehrlichia mikurensis*, *Rickettsia* spp., and epizootic haemorrhagic disease virus (EHDV), and by commercial enzyme-linked immunosorbent assay (ELISA) for antibodies against bluetongue virus (BTV). The possible associations of host factors and density with pathogen prevalence and co-infection, and in the case of *A. phagocytophilum* with bacterial load, were assessed using generalized linear modelling.

**Results and conclusion:**

Analysis revealed the following prevalence in roe deer: *A. phagocytophilum* 77.9%, *Bartonella* spp. 77.7%, *Babesia* spp. 17.4%, *Rickettsia* spp. 3.3%, *B. burgdorferi* sensu lato 0.2%. Various co-infections were found, of which *A. phagocytophilum* and *Bartonella* spp. (49.7% of infected roe deer) and *A. phagocytophilum*, *Bartonella* spp. and *Babesia* spp. (12.2% of infected roe deer) were the most common. *Anaplasma phagocytophilum*, *Babesia* spp., and co-infection prevalence were significantly higher in calves than in adult roe deer, whereas the prevalence of *Bartonella* spp. was lower in roe deer in good nutritional condition than in deer in poor nutritional condition. Local roe deer density was not associated with pathogen presence. The high prevalence of *A. phagocytophilum*, *Bartonella* spp., and *Babesia* spp. is evidence for the role of roe deer as reservoirs for these pathogens. Additionally, the results suggest a supportive role of roe deer in the life-cycle of *Rickettsia* spp. in the Netherlands.

**Graphical Abstract:**

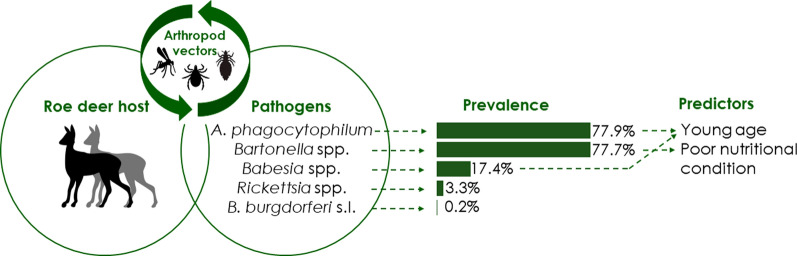

**Supplementary Information:**

The online version contains supplementary material available at 10.1186/s13071-022-05195-w.

## Background

Since the 1990s, several vector-borne diseases have been emerging in Europe in both humans and animals [1–4]. This trend is apparent for enzootic diseases such as Lyme borreliosis in humans caused by the tick-borne pathogen *Borrelia burgdorferi* sensu lato (s.l.), as well as for novel vector-borne diseases such as bluetongue in livestock induced by a virus transmitted via *Culicoides* midges [5, 6]. Wildlife species affect the emergence of vector-borne diseases by being more or less competent hosts for vectors and the vector-borne pathogens these transmit [7]. One of these wildlife host species is the roe deer (*Capreolus capreolus*). Roe deer is the most numerous cervid species in the Netherlands (estimated > 60,000 in 2008), found throughout the country though predominantly in the eastern part [8].

Roe deer are competent hosts for the vector-borne pathogens *Anaplasma phagocytophilum,* some *Bartonella* species and a few *Babesia* species. Reported *A. phagocytophilum* prevalence in roe deer ranges from 9.6% in Poland to 98.9% in Germany [9–17]. Roe deer are mostly infected with the non-zoonotic *A. phagocytophilum* ecotype II, rarely with the zoonotic ecotypes I and III [16, 18, 19]. The main vector of *A. phagocytophilum* in Europe is *Ixodes ricinus* (Acari: Ixodidae, sheep tick) [13, 20], in which no transovarial transmission has been demonstrated to date [21, 22]. Between 0 and 10.6% prevalence was reported in questing *I. ricinus* in the Netherlands [19, 23]. The *Bartonella* species found in roe deer include *Bartonella capreoli,* the zoonotic *Bartonella schoenbuchensis* and *Bartonella bovis* [24–26]. *Bartonella* spp. infection rates reported in roe deer blood samples from Poland ranged between 13.4 and 27.6% [11, 26]. In Germany, *B. schoenbuchensis* was isolated from four out of five roe deer [27]. Potential vectors of these bacteria include ticks and keds [24, 28]. The deer ked *Lipoptena cervi* (Hippoboscidae) is the main vector of *B. schoenbuchensis* [29]. The two most commonly detected *Babesia* species in roe deer are *Babesia capreoli* and, to a lesser extent, the zoonotic *Babesia venatorum* (previously named *Babesia* sp. EU1) [10, 17, 30–32]. *Babesia divergens* was occasionally reported in earlier studies, but this could have been *Babesia capreoli* [31, 33–35]. Incidentally, *Babesia bigemina*, *Babesia* sp. MO1 and *Babesia microti*-like species have also been reported in roe deer [32, 35, 36]. Reported *Babesia* spp. prevalence in roe deer in Europe ranges from 8.7% to 89.5% [10, 17, 35, 37, 38]. *Ixodes ricinus* is a primary vector of *Babesia capreoli* and *B. venatorum* in Europe [39, 41]. Transstadial and transovarial transmission occurs for *B. venatorum*, but only transstadial for *B. microti* [30, 41]*.*

Roe deer are considered to be incompetent hosts for *B. burgdorferi* s.l. [42, 43] because of the borreliacidal activity of their innate immune system [42, 43], and are possibly also incompetent hosts for *Borrelia miyamotoi*, the causative agent of relapsing fever in humans [44]. Although they seroconvert upon *B. burgdorferi* s.l. exposure through tick bites [15], deer are not infective to feeding *I. ricinus* ticks [42]. The role of roe deer in enhancing the emergence of Lyme borreliosis has been associated with their importance as a propagation host for *I. ricinus* rather than as a reservoir of the pathogen [2, 5]. In addition, roe deer are unlikely to be an important reservoir of *Neoehrlichia mikurensis.* Limited research on potential host species of *N. mikurensis* suggests that rodents are the natural reservoir [45, 46]. *Ixodes ricinus* is the vector of these three pathogens, with *B. burgdorferi* s.l. being detected in 11.8% of the questing nymphs and adults in the Netherlands [23], *B. miyamotoi* in 2.9% [44] and *N. mikurensis* in 5.6–11% [23, 47].

The competence of roe deer as hosts of *Rickettsia* spp. causing spotted fever syndrome is unclear [48]. Blood samples from 4 out of 21 (19%) roe deer from the period 2000–2002 in the Netherlands tested positive for *Rickettsia helvetica* [48]. These four deer were not reported to have clinical signs [48]. *Rickettsia helvetica* is one of the two main spotted fever *Rickettsia* transmitted by *I. ricinus* in Europe, with tick infection rates in the Netherlands ranging from 6 to 66% [48].

Roe deer can be infected with the *Culicoides*-borne orbiviruses bluetongue virus (BTV) and epizootic haemorrhagic disease virus (EHDV) [6, 49, 50]. BTV seroprevalence studies in Spain, France and Belgium showed that after BTV introduction, antibodies were present in 1.2% (France) up to 5.1% (Spain) of the roe deer [6, 49]. BTV is not enzootic in the Netherlands, but an epidemic (BTV-8 epidemic 2006–2008) and incidental (BTV-6 in 2008) introductions have been recorded in livestock [6]. Roe deer have shown no clinical signs but had viraemia after being experimentally infected with EHDV [50]. The viraemia was lower in roe deer than in other cervid species [50]. There are no known incursions of EHDV into Europe, but the disease is present in cattle in North Africa and the Middle East [51].

To date, no studies have investigated the prevalence of these nine vector-borne pathogens or pathogen genera (for simplicity referred to as pathogens from this point forward) in Dutch roe deer. The aim of this cross-sectional study was therefore to determine the apparent prevalence and co-infection of these nine vector-borne pathogens in roe deer blood in the Netherlands and to identify which host variables predict their presence in roe deer.

## Methods

### Cross-sectional blood sampling of roe deer

Blood samples were collected during the doe-and-calf hunting season (December 2009 to March 2010) and the buck hunting season (April 2010 to September 2010). Sampling packages were assigned to game management units (GMUs) at the beginning of each of these two hunting seasons (500 in December 2009; 200 in April 2010), using a random sampling scheme based on the number of roe deer counted in total (*n* = 57,264) and per GMU in 2008 and on the overall composition of this count (44% does and 26% calves, 30% bucks). Hunters were requested to collect blood samples post-mortem from the vessels in the neck, axilla or inguina, the heart, the chest cavity or abdominal cavity of hunted roe deer, by using serum and ethylenediaminetetraacetic acid (EDTA)-blood syringes of the S-Monovette collection system (Sarstedt, Nümbrecht, Germany) without needles. Moreover, the hunters noted information regarding the approximate age of the animals (calf, yearling, adult), sex (female, male), nutritional condition (poor, moderate, good), health status (ill health, healthy) and the exact weight (indicating if weighed with or without head and or legs). A calf is a deer < 1 year of age, a yearling is between 1 and 2 years old, and adult roe deer are deer older than 2 years. This information was sent with the blood samples to the Dutch Wildlife Health Centre, where the samples were divided into aliquots and kept frozen (−80 °C) until further processing. In total, 461 (65.9%) samples were returned and suitable for pathogen detection: 344 obtained in the doe-and-calf hunting season (return rate 68.8%, 344/500), and 117 in the buck hunting season (return rate 58.5%, 117/200).

### Detection of vector-borne bacteria and protozoa

DNA from blood samples was extracted using the DNeasy^®^ Blood & Tissue kit (QIAGEN, Hilden, Germany) as per the manufacturer’s instructions. To detect potential cross-contamination negative controls were included in each batch of extraction. Samples were tested with quantitative polymerase chain reaction (qPCR) for the presence of *A. phagocytophilum* [19], *B. burgdorferi* s.l. [52], *B. miyamotoi* [53], spotted fever *Rickettsia* [54] and *N. mikurensis* [47]. For detection of *Babesia* spp. a conventional PCR assay was used, which targets a fragment of the *Babesia* 18S rDNA of several *Babesia* spp. [55]. *Bartonella* spp. DNA was detected by PCR as previously described [28]. The PCR products were analysed with gel electrophoresis on a 1.5% agarose gel and coloured with SYBR™ Gold Nucleic Acid Gel Stain (Invitrogen, Carlsbad, CA, USA). Positive controls and negative water controls were used on every plate tested. To minimize contamination and false-positive samples, the DNA extraction, PCR mix preparation, sample addition and (q)PCR analyses were performed in separate air-locked dedicated labs. Sequencing of positive *Bartonella* and *Babesia* samples followed conventional PCR for species identification, i.e., for *Bartonella*, DNA sequencing of a ~ 380-base-pair (bp) fragment of the citrase synthase gene [56], and for *Babesia*, sequencing of a ~ 400 bp fragment of the 18S rDNA [55]. *Bartonella* and *Babesia* species identification was determined using BLAST (Basic Local Alignment Sequencing Tool), and by using Bionumerics 7.5 for comparisons to in-house molecular databases and to sequences obtained from NCBI.

### Detection of vector-borne viruses

For the detection of EHDV RNA, 125 µl blood was diluted to 250 µl, from which 200 µl was then taken to extract genetic material using the MagNAPure Total NA isolation kit (Roche Diagnostics GmbH, Mannheim, Germany). The genetic material was extracted in 50 µl of RNAse-free water, of which 5 µl was then pooled with that of four other samples for use in the PCR test. If positive, the samples of that pool would then be tested separately. The PCR test was a modified real-time RT-PCR test based on the Kit TaqVet^®^ epizootic haemorrhagic disease virus kit (LSI, Lissieu, France). A commercial ELISA was used to detect BTV-reactive antibodies in roe deer serum (or plasma) samples (IDvet, Montpellier, France). Samples from the doe-and-calf hunting season would be tested first for these two orbiviruses and those from the buck hunting season only if samples from the doe-and-calf hunting season tested positive.

### Statistical analyses

#### Sample characteristics

Host factor categories were plotted per month for insight into their temporal distribution, and count differences in host factors per hunting season were investigated using the Chi-square test (χ^2^) or the Fisher’s exact test. A Chi-square test was also performed for comparison of the proportions of calves (female calves and male calves), does (yearling and adult females) and bucks (yearling and adult males) in the sample to those in the counted roe deer population.

The spatial distribution of the samples was mapped against five roe deer density classes in GMUs using ArcGIS 10.5.1 (Esri, 2017): 0; > 0 and ≤ 2; > 2 and ≤ 4; > 4 and ≤ 6; and > 6 roe deer per 100 ha. GMU roe deer density was calculated by dividing the number of roe deer counted in the GMU in 2008 by the surface of the GMU; both the nominator and the denominator were provided by the Royal Dutch Hunters’ Association (KNJV). To investigate whether the spatial distribution of the samples was significantly related to GMU deer density, a linear regression (LR) model was fitted to quantify the effect of GMU roe deer density on the number of samples supplied by the different GMUs. GMUs where no roe deer were counted or where hunting was not authorized were removed (76/318) in advance. The mean density of the roe deer in the sample was compared to the mean density of roe deer in the 242 GMUs by *t*-test, and if different, investigated further by comparing density class distributions.

The correspondence of body mass to the four roe deer host factors and density was examined by fitting a multiple linear regression (MLR) model to quantify the effect of these parameters on body mass, using ‘calf’, ‘female’, ‘poor nutritional condition’ and ‘ill-health’ as reference categories for the categorical data. The sign of the estimate and statistical significance of each host factor in the expected direction for body mass was used as global verification of the host factor data submitted by the hunters.

#### Pathogen (sero-)prevalence, spatio-temporal distributions and co-infection

Apparent prevalence was determined for *A. phagocytophilum*, *Babesia* spp., *Bartonella* spp., *B. burgdorferi* s.l., *B. miyamotoi,* EHDV, *N. mikurensis*, *Rickettsia* spp. and seroprevalence for BTV. Diagnostic test results were used to generate presence and absence maps per pathogen and hunting season distributions.

Subsequently, pathogen co-infections were quantified. A two-by-two comparison of pathogens using the χ^2^ test or Fisher’s exact test was performed to assess whether the number of co-infections observed could be explained by chance. For the determination of the association between the presence of a given pathogen and the presence of the other investigated pathogens, a generalized linear model (GLM) was performed, with a binomial distribution in regard to pathogen presence or not.

#### Association of pathogens with roe deer host factors and density

For the determination of the association between the presence of vector-borne pathogens and the host factors (age category, sex, nutritional condition, health status) and density, a GLM regression was performed. If relevant, a second GLM model was fitted to obtain the effect of host factors and density on pathogen load (C_q_-value). Because no reference DNA was used to validate each individual C_q_-value, C_q_-value was included as binary response variable with an arbitrary cut-off value of 30 (1 if C_q_-value ≤ 30; 0 if C_q_-value > 30). Similar analyses were performed for the presence of co-infections in relation to host factors and density (GLM), and for the effect of host factors and density on the number of different co-infecting pathogens present in roe deer.

All statistical analyses were conducted in R (R version 4.0.0 [2020-04-24]). Predictors were selected by backward stepwise selection, using the Akaike information criterion (AIC) value for selection of the model with the best fit. Models were run using subsets of data with values for the dependent and all the predictor variables. The retained models were checked for multicollinearity based on the variation inflation factor (VIF), normality and homoscedasticity. The level of statistical significance in all tests was set at *P* = 0.05. The databases and R-script are available as supplementary material (Additional files 1, 2 and 3).

## Results

### Sample characteristics and identification of possible sources of bias

**Fig. 1:**
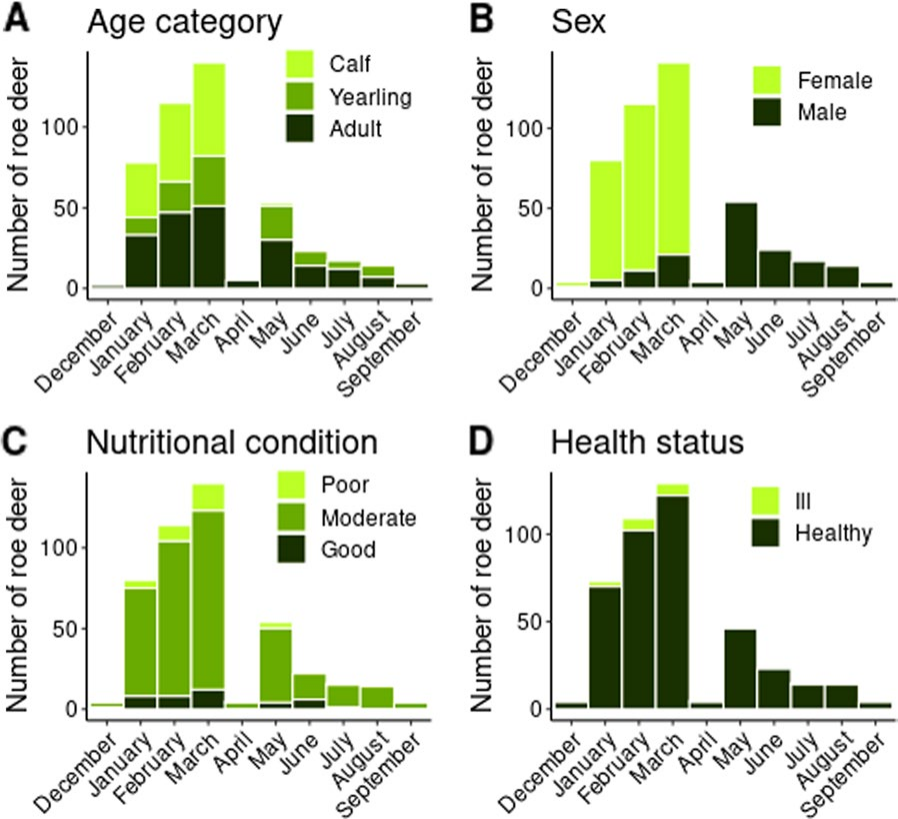
Distribution of the sampled roe deer per host factor per month. The doe-and-calf hunting season is from December to March, the buck hunting season from April to September. The distributions of age category and sex are significantly affected by the hunting season, but the distributions of nutritional condition and health status are not

The blood samples were obtained from 461 roe deer of different age categories (145 calf, 105 yearling, 204 adult, 7 not available; NA), sex (304 female, 155 male, 2 NA), nutritional condition (38 poor, 376 moderate, 40 good, 7 NA) and health status (404 healthy, 19 ill, 38 NA) (Additional file 1: sample database). Age and sex categories expectedly showed a pattern consistent with the differential hunting seasons (Fig. 1a, Chi-square test for age category, χ^2^ = 63.36, *df* = 2, *P* < 0.001; Fig. 1b, Chi-square test for sex, χ^2^ = 304.01, *df* = 1, *P* < 0.001). No significant differences were detected between the two hunting seasons in the nutritional condition (Fig. 1c; Chi-square test, χ^2^ = 3.19, *df* = 2, *P* = 0.203) or in health status (Fig. 1d*;* Fisher’s exact test, *P* = 0.054, odds ratio [OR] = 6.3, 95% confidence interval [CI] = 1–265). Compared to the proportions in the counted population (26% calves, 44% does, 30% bucks), the proportion of calves in the sample was slightly overrepresented (31.9%, 144/452) at the cost of bucks (26.3%, 119/452) and to lesser extent does (41.8%, 189/452) (Chi-square test, χ^2^ = 8.49, *df* = 2, *P* = 0.014).

**Fig. 2:**
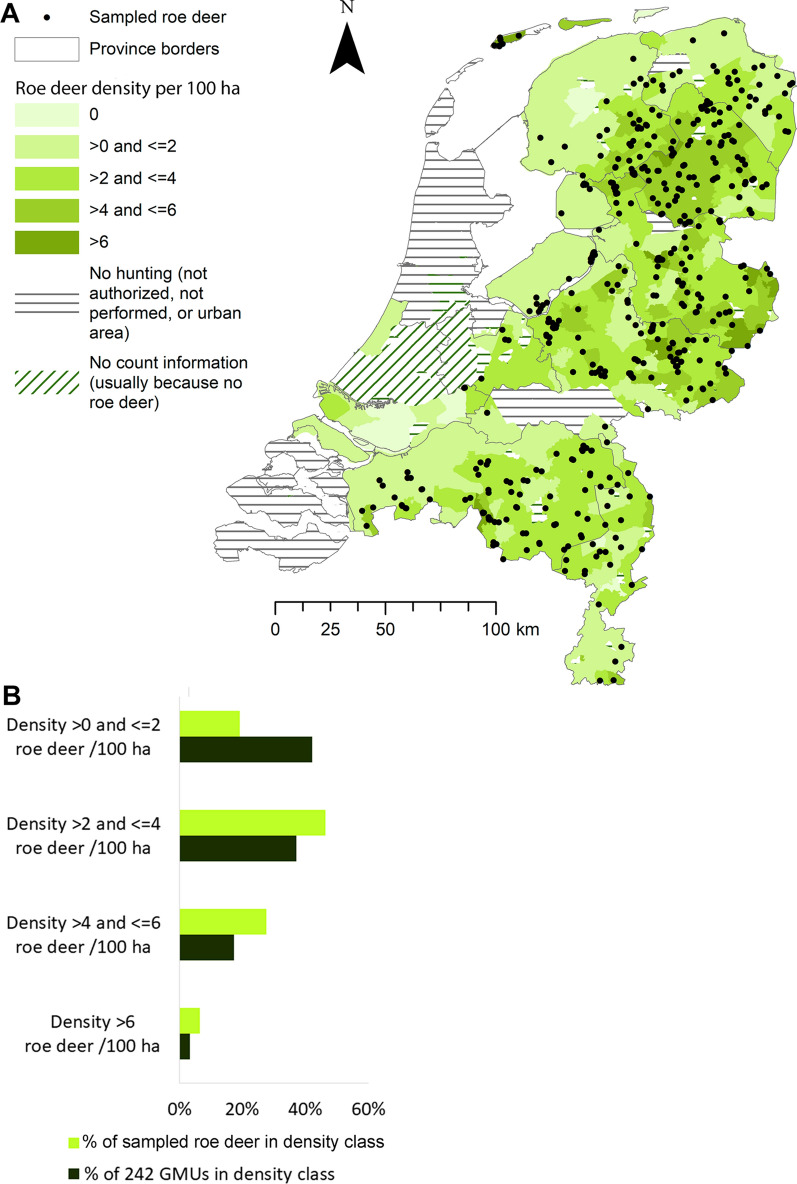
Spatial distribution of the sampled roe deer in relation to roe deer density. **a** Sample distribution over GMU roe deer density. **b** The plot of the density category distributions of sample and GMUs demonstrates an underrepresentation of roe deer from GMUs with ≤ 2 roe deer per 100 ha in the sample

The spatial distribution of the samples mapped against roe deer density classes is shown in Fig. 2a. Roe deer densities in the 242 GMUs ranged from 0.06 to 10.36 roe deer per 100 ha (Additional file 2: GMU database). The number of samples provided by a GMU significantly increased with GMU roe deer density (estimate ± SE = 0.544 ± 0.092; *F*_(1, 240)_ = 34.93, *P* < 0.001), but the model had a poor fit (adjusted *R*^2^ = 12.3%). The mean ± SD roe deer density per 100 ha in the sampled population (3.41 ± 1.74) was significantly higher than the mean for the 242 GMUs (2.55 ± 1.74; *t*-test, *t*_(490)_ = 6.248, *P* =  < 0.001). The density category proportions differed significantly between sample and the 242 GMUs (Chi-square test, χ^2^ = 43.73, *df* = 3, *P* < 0.001), with an underrepresentation of roe deer from GMUs with ≤ 2 roe deer per 100 ha in the sample (Fig. 2b).

Body weight ranged from 7.0 kg to 22.0 kg for females and from 7.0 kg to 23.0 kg for males. The model for body weight had a good fit (adjusted *R*^2^ = 67.2%) and maintained all five investigated host variables, with body weight being significantly positively associated with increasing age, male sex, better nutritional condition and healthy status, and significantly negatively associated with increasing roe deer density (Table 1). Details on mean body weight per sex-age category are provided as supplementary information (Additional file 4).

**Table 1:** Host factor and density predictors of roe deer body weight (*n* = 329)

Dependent variable	Model structure^a^	Estimate ± SE	*t*	*P*
Roe deer body weight	Intercept	7.549 (0.513)	14.705	< 0.001*
	Age category—yearling	2.845 (0.280)	10.167	< 0.001*
	Age category—adult	4.783 (0.231)	20.691	< 0.001*
	Sex—male	0.776 (0.213)	3.645	< 0.001*
	Nutritional condition—moderate	2.151 (0.467)	4.602	< 0.001*
	Nutritional condition—good	3.875 (0.568)	6.820	< 0.001*
	Health status—healthy	1.584 (0.584)	2.713	0.007*
	Density	−0.164 (0.056)	−2.916	0.004*

^a^Predicted effect of age category (calf, yearling, adult), sex (female, male), nutritional condition (poor, moderate, good) and health (ill, healthy) on the variable body weight, subset data (*n* = 329). Model reference categories: age category: calf; sex: female; nutritional condition: poor; health status: ill

*Statistically significant

### Pathogen (sero-)prevalence, spatio-temporal distributions and co-infection

**Fig. 3:**
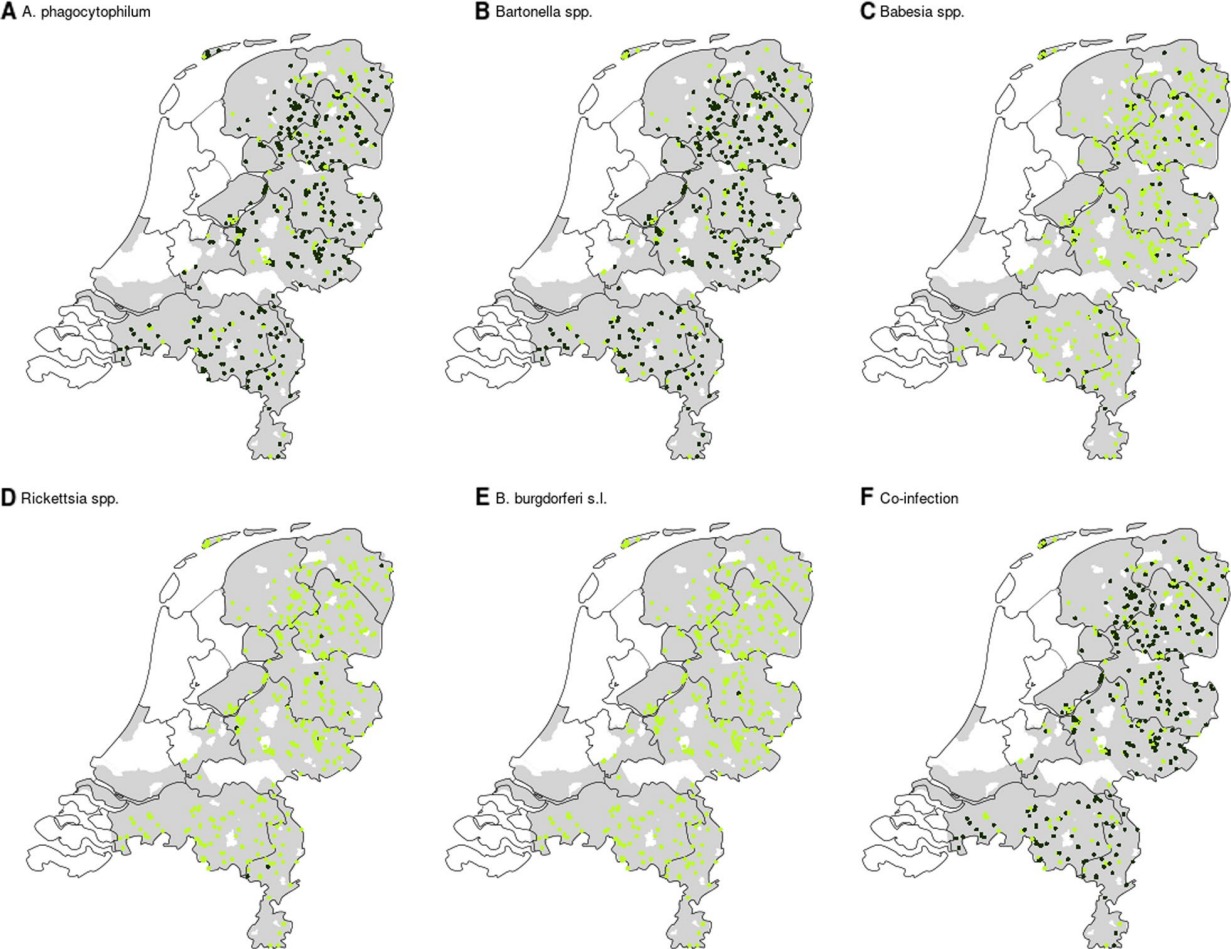
Presence and absence of vector-borne pathogens and co-infection in the Netherlands (*n* = 461)*.* **a** Sample distribution of *A. phagocytophilum*. **b** Sample distribution of *Bartonella* spp. **c.** Sample distribution of *Babesia* spp. **d** Sample distribution of *Rickettsia* spp. **e** Sample distribution of *B. burgdorferi* s.l. **f** Presence of co-infection. The dark green dots represent vector-borne pathogen presence, and the light dots represent vector-borne pathogen absence

Genetic material of at least one of the investigated pathogens was detected in a high proportion of the roe deer sampled (92.0%, 424/461) (Table 2). DNA of *A. phagocytophilum* was detected in 77.9% (95% CI 73.7–81.5) of the samples, *Bartonella* spp. in 77.7% (95% CI 73.5–81.3), *Babesia* spp. in 17.4% (95% CI 14.1–21.2) and *Rickettsia* spp. in 3.3% (95% CI 1.9–5.4) (Table 2). Positive samples occurred widespread throughout the country in the roe deer population (Fig. 3). There were no significant temporal differences except for *Babesia* spp., which was less frequently detected in the buck hunting season (Chi-square test, χ^2^ = 9.32, *df* = 1, *P* = 0.002). Only one animal tested positive for *B. burgdorferi* s.l. by qPCR. Several attempts to confirm the presence of *B. burgdorferi* s.l. DNA by conventional PCR of a small fragment of the intergenic spacer region were unsuccessful [57]. None of the 461 roe deer tested positive for the presence of *B. miyamotoi* or *N. mikurensis* DNA*.* In addition, none of the roe deer sampled in the doe-and-calf period tested positive for EHDV RNA (*n* = 344) or showed evidence for BTV antibodies (*n* = 338; 6 NA).

**Table 2:** Prevalence of single pathogen infections and different combinations of two- and three-pathogen co-infections (*n* = 461)

No. of pathogens	Pathogen species	No. of roe deer (%)
0	–	37 (8.0%)
1	*Rickettsia*	1 (0.2%)
	*Babesia*	2 (0.4%)
	*Anaplasma*	49 (10.6%)
	*Bartonella*	53 (11.5%)
	**Subtotal**	105 (22.8%)
2	*Bartonella* & *Rickettsia*	1 (0.2%)
	*Anaplasma* & *Rickettsia*	3 (0.7%)
	*Bartonella* & *Babesia*	8 (1.7%)
	*Anaplasma* & *Babesia*	11 (2.4%)
	*Anaplasma* & *Bartonella*	229 (49.7%)
	**Subtotal**	252 (54.7%)
3	*Anaplasma* & *Bartonella* & *Borrelia*	1 (0.2%)
	*Anaplasma* & *Bartonella* & *Rickettsia*	7 (1.5%)
	*Anaplasma* & *Bartonella* & *Babesia*	56 (12.2%)
	**Subtotal**	64 (13.9%)
4	*Anaplasma* & *Bartonella* & *Babesia* & *Rickettsia*	3 (0.7%)

DNA sequencing a ~ 380 bp fragment of the citrase synthase gene from 47/358 *Bartonella* spp. positive samples indicated the presence of *B. schoenbuchensis* (21/47, 44.7%) and *Bartonella capreoli* (5/47, 10.6%), while unsuccessful for 21 samples (21/47, 44.7%). The *B. schoenbuchensis* sequences were more than 99% similar to *B. schoenbuchensis* found in Germany (Genbank accession numbers AJ564632 and AJ564633). The five *Bartonella capreoli* sequences were more than 98% similar to the *Bartonella capreoli* found in roe deer from Poland (JQ929915) and France (AF293392). Sequencing a ~ 400 bp fragment of the 18S rDNA of the 80 *Babesia* positive samples showed the presence of *Babesia capreoli* (27/80, 33.75%) and *B. microti* (3/80, 3.75%), while unsuccessful in 50 samples (50/80, 62.5%). The *Babesia capreoli* sequences were more than 99% similar to the *Babesia capreoli* from roe deer (AY726009) or *I. ricinus* (FJ215873) from France. The three *B. microti* sequences were more than 99.5% similar to the *B. microti* found in *I. ricinus* from Switzerland (AF494286) and Slovenia (AF373332). Sequences from this study can be found in Additional file 5.

Vector-borne pathogen co-infection was detected in 69.2% (319/461) of the samples. The most common co-infection combinations observed in the sample were *A. phagocytophilum* and *Bartonella* spp. and *A. phagocytophilum*, *Bartonella* spp. and *Babesia* spp. (Table 2). All other combinations each represented only small percentages (Table 2). Co-infections involving *A. phagocytophilum* and *Bartonella* spp. occurred more frequently than could be expected by chance, as did co-infections involving *A. phagocytophilum* and *Babesia* spp. (Table 3). The positive associations of *A. phagocytophilum* with *Bartonella* spp. and *Babesia* spp., of *Bartonella* spp. with *A. phagocytophilum,* and of *Babesia* spp. with *A. phagocytophilum* were further confirmed by the GLM results (Table 4).

**Table 3:** Probability of co-infections occurring by chance (*n* = 461)

	*Bartonella* spp.	*Babesia* spp.	*Rickettsia* spp.
*A. phagocytophilum*	O^a^: 296 (64.2%)	O: 70 (15.2%)	O: 13 (2.8%)
	E^b^: 278.79 (60.5%)	E: 62.29 (13.5%)	E:11.68 (2.5%)
	*χ*^2^ = 20.26, *df* = 1, *P* < 0.001* ^c^	*χ*^2^ = 4.55, *df* = 1, *P* = 0.033*	*P* = 0.539, OR = 1.9, 95% CI = 0–17
*Bartonella* spp.		O: 67 (14.5%)	O: 11 (2.4%)
		E: 62.13 (13.5%)	E: 11.65 (2.5%)
		χ^2^ = 1.67, *df* = 1, *P* = 0.197	*P* = 0.752, OR = 0.78, 95% CI = 0–3
*Babesia* spp.			O: 3 (0.65%)
			E: 2.6 (0.56%)
			*P* = 0.732, OR = 0.78, 95% CI = 0–3

^a^Observed

^b^Expected

^c^*P*-value as determined by Chi-square test (*A. phagocytophilum, Babesia* spp., *Bartonella* ssp.) or Fisher’s exact test (*Rickettsia* spp.)

*Statistically significant

**Table 4:** Co-infecting pathogen predictors for the investigated pathogens (*n* = 461)

Dependent variable	Model structure^a^	Estimate (± SE)	*z*	*P*	Odds ratio (95% CI)
*A. phagocytophilum*	Intercept	0.370 (0.207)	1.791	0.073	1.4 (1.0–2.2)
	*Bartonella* spp.	1.081 (0.247)	2.018	< 0.001*	2.9 (1.8–4.8)
	*Babesia* spp.	0.735 (0.364)	4.374	0.044*	2.1 (1.1–4.5)
*Bartonella* spp.	Intercept	0.438 (0.203)	2.161	0.031*	1.5 (1.0–2.3)
	*A. phagocytophilum*	1.109 (0.246)	4.513	< 0.001*	3.0 (1.9–4.9)
*Babesia* spp.	Intercept	−2.219 (0.333)	−6.666	< 0.001*	0.1 (0.1–0.2)
	*A. phagocytophilum*	0.801 (0.359)	2.234	0.026*	2.2 (1.1–4.8)

^a^Predicted effect of other pathogens on prevalence *A. phagocytophilum*, *Bartonella* spp., *Babesia* spp. and *Rickettsia* spp

*Statistically significant

### Pathogen associations with roe deer host factors and density

The prevalence of pathogens per host factor category is summarized in Table 5. The presence of *A. phagocytophilum* DNA was associated with age category only, with adult roe deer having significantly lower odds of testing positive than calves (Table 6). A C_q_-value ≤ 30 was best predicted by a model including age category and sex, the odds significantly decreasing with being adult and increasing with being male (Table 7).

**Table 5:** Prevalence of the four most prevalent vector-borne pathogens in roe deer by host age category, sex, nutritional condition and health status

Host factor	Host factor category (sample size)	Pathogen prevalence (%) (95% CI)			
		*A. phagocytophilum*	*Bartonella* spp.	*Babesia* spp.	*Rickettsia* spp.
Age category	Calf (* n* = 145)	85.5 (78.5–90.6)	78.6 (70.9–84.8)	25.5 (18.8–33.5)	3.4 (1.3–8.3)
	Yearling (* n* = 105)	77.1 (67.7–84.5)	77.1 (67.7–84.5)	15.2 (9.2–23.9)	5.7 (2.3–12.5)
	Adult (* n* = 204)	73.0 (66.3–78.8)	77.5 (71.0–82.9)	12.7 (8.6–18.3)	1.5 (0.4–4.6)
Sex	Female (* n* = 304)	76.6 (71.4–81.2)	76.0 (70.7–80.6)	19.1 (14.9–24.0)	3.6 (1.9–6.6)
	Male (* n* = 155)	80.6 (73.4–86.3)	80.6 (73.4–86.4)	14.2 (9.3–20.9)	2.6 (0.8–6.9)
Nutritional condition	Poor (* n* = 38)	73.7 (56.6–86.0)	86.8 (71.1–95.0)	15.8 (6.6–31.9)	10.5 (3.4–25.7)
	Moderate (* n* = 376)	79.3 (74.7–83.1)	77.6 (73.0–81.7)	17.8 (14.2–22.1)	2.7 (1.4–5.0)
	Good (* n* = 40)	67.5 (50.8–80.9)	70.0 (53.3–82.9)	10.0 (3.3–24.6)	2.5 (0.1–14.7)
Health status	Ill (* n* = 19)	73.7 (48.6–89.9)	84.2 (59.5–95.8)	31.6 (13.6–56.5)	10.5 (1.8–34.5)
	Healthy (* n* = 404)	78.2 (73.8–82.1)	77.9 (73.5–81.9)	16.8 (13.4–20.9)	2.7 (1.4–5.0)

**Table 6:** Host factor and density predictors for the investigated pathogens and co-infection (*n* = 409)

Dependent variable	Final model^a^	Estimate (± SE)	*z*	*P*	Odds ratio (95% CI)
*A. phagocytophilum*	Intercept	1.774 (0.248)	7.150	< 0.001*	5.9 (3.1–9.9)
	Age category—yearling	−0.602 (0.348)	−1.731	0.083	0.5 (0.3–1.1)
	Age category—adult	−0.725 (0.300)	−2.422	0.015*	0.5 (0.3–0.9)
*Bartonella* spp.	Intercept	2.234 (0.607)	3.677	< 0.001*	9.3 (3.3–39.0)
	Nutritional condition—moderate	−0.971 (0.621)	−1.563	0.118	0.4 (0.1–1.1)
	Nutritional condition—good	−1.336 (0.705)	−1.895	0.058	0.3 (0.1–0.9)
*Babesia* spp.	Intercept	−0.270 (0.527)	−0.513	0.608	0.8 (0.3–2.1)
	Age category—yearling	−0.741 (0.361)	−2.053	0.040*	0.5 (0.2–0.9)
	Age category—adult	−0.862 (0.297)	−2.901	0.004*	0.4 (0.2–0.8)
	Health status—healthy	−0.830 (0.523)	−1.586	0.113	0.4 (0.2–1.3)
*Rickettsia* spp.	Intercept	−2.178 (0.884)	−2.463	0.014*	0.1 (0.0–0.5)
	Age category—yearling	1.220 (0.737)	1.654	0.098	3.4 (0.8–16.9)
	Age category—adult	−0.359 (0.830)	−0.433	0.665	0.7 (0.1–3.9)
	Health status—healthy	−1.796 (0.848)	−2.118	0.034*	0.2 (0.0–1.2)
Co-infection	Intercept	1.171 (0.206)	5.697	< 0.001*	3.2 (2.2–4.9)
	Age category—yearling	−0.429 (0.302)	−1.419	0.156	0.7 (0.4–1.2)
	Age category—adult	−0.534 (0.257)	−2.077	0.038*	0.6 (0.4–1.0^b^)

^a^Final models on predicted effect of host traits and density on detection of the pathogens *A. phagocytophilum*, *Bartonella* spp., *Babesia* spp., *Rickettsia* spp. and the presence of co-infection in roe deer blood samples

^b^The upper limit of this 95% CI is 0.964, i.e., 95% CI excludes 1

*Statistically significant

**Table 7:** Host factor and density predictors for *A. phagocytophilum* C_t_ load < 30 (*n* = 359)

Dependent variable	Model structure^a^	Estimate (± SE)	*z*	*P*	Odds ratio (95% CI)
*A. phagocytophilum*	Intercept	−0.349 (0.201)	−1.738	0.082	0.7 (0.5–1.0)
	Age category—yearling	−0.535 (0.323)	−1.657	0.097	0.6 (0.3–1.1)
	Age category—adult	−0.574 (0.268)	−2.142	0.032*	0.6 (0.3–1.0^b^)
	Sex—male	0.549 (0.249)	2.206	0.027*	1.7 (1.1–2.8)

^a^ Predicted effect of age category (calf, yearling, adult) and sex (female, male) for *A. phagocytophilum* C_t_ load, subset data (*n* = 359). Model reference categories: age category: calf; sex: female

^b^ The upper limit of this 95% CI is 0.95, i.e., 95% CI excludes 1

*Statistically significant

The presence of *Bartonella* spp. was best predicted by a model with only nutritional condition. While not statistically significant, the odds of testing positive for *Bartonella* spp. were lower in roe deer in good condition compared to those in poor condition (Table 6).

The presence of *Babesia* spp*.* was best predicted by a model including the age category and health, with both yearling and adult animals having significantly lower odds of testing positive than calves (Table 6).

The presence of *Rickettsia* spp*.* was best predicted by a model including age category and health. Despite a *P*-value suggesting significantly lower odds for *Rickettsia* spp. in healthy compared to ill roe deer, this effect of the factor health was not substantiated by the confidence interval (Table 6).

The occurrence of co-infection was associated with the host age category, with adult roe deer having significantly lower odds of having co-infections than calves (Table 6). The number of co-infecting pathogens was best predicted by a model including age category and health, with a significant negative effect of being adult (Table 8).

**Table 8:** Host factor and density predictors for the number of co-infecting pathogens (*n* = 284)

Dependent variable	Model structure^a^	Estimate (± SE)	*t*	*P*	Odds ratio (95% CI)
Number of co-infecting pathogens^b^	Intercept	2.531 (0.119)	21.292	< 0.001*	12.6 (9.9–15.9)
	Age category—yearling	−0.110 (0.069)	−1.600	0.111	0.9 (0.8–1.0^c^)
	Age category—adult	−0.173 (0.058)	−3.006	0.003*	0.8 (0.8–0.9)
	Health status—healthy	−0.222 (0.117)	−1.899	0.059	0.8 (0.6–1.0^d^)

^a^Predicted effect of age category (calf, yearling, adult) and health (ill, healthy) on the number of co-infecting pathogens, subset data (*n* = 284). Model reference categories: age category: calf; health status: ill

^b^*R*^2^ = 0.033, *P* = 0.006

^c^The upper limit of this 95% CI is 0.1.026, i.e., 95% CI includes 1

^d^The upper limit of this 95% CI is 0.1.008, i.e., 95% CI includes 1

*Statistically significant

## Discussion

In this cross-sectional study from 2010, five of the nine investigated vector-borne pathogens were detected in the Dutch roe deer population. Roe deer harboured *A. phagocytophilum*, *Bartonella* spp., *Babesia* spp., *Rickettsia* spp. and *B. burgdorferi* s.l. There was no evidence for infection with *B. miyamotoi*, *N. mikurensis* or EHDV, nor was there any serological evidence for exposure to BTV. Most of the vector-borne pathogen-positive roe deer were co-infected with both *A. phagocytophilum* and *Bartonella* spp., followed by triple infection with *A. phagocytophilum*-*Bartonella* spp.-*Babesia* spp. The prevalence of *A. phagocytophilum*, *Babesia* spp. and co-infections was highest in calves. The prevalence of *Bartonella* spp. was highest in roe deer in poor nutritional condition.

The DNA of *A. phagocytophilum* was found in 77.9% of the samples. This relatively high prevalence supports a primary role of roe deer in the maintenance of *A. phagocytophilum* in the Netherlands [58]. However, this is presumably the commonly detected non-zoonotic ecotype II [16, 18, 19, 59, 60]. *Anaplasma phagocytophilum* causes disease by infecting phagocytic cells, mainly neutrophils, in which it replicates and spreads to tissues [20]. Current knowledge indicates that infection is often subclinical, but clinical disease can occur, in particular in young or naïve animals [20, 61]. The odds of detecting *A. phagocytophilum* were significantly greater in calves than in adult animals. This suggests *A. phagocytophilum* infection may be less present in animals > 2 years old. Alternatively, some adults may have acquired premunition with a level of infection that is reduced to below the limit of detection by PCR. Finding that C_q_-value ≤ 30 (high pathogen load) negatively associated with older age indicates lower *A. phagocytophilum* loads in adult roe deer and supports premunition in mature animals. Prevalence of *A. phagocytophilum* did not differ between sexes, but the quantity of *A. phagocytophilum* DNA tended to be greater in positive samples from males compared to females. This suggests no sex difference in minimal exposure for detectable infection but rather points to a greater intensity level of pathogen exposure, reduced immunocompetence, or both in males. Due to the differential hunting seasons, males were sampled mostly when there was greater tick activity [62] and during mating season. Mating has been associated with reduced immunological defences in male ungulates [63]. Previous studies found no association between *A. phagocytophilum* prevalence and age and sex of hosts [10, 14, 15, 17, 18].

*Bartonella* spp. DNA was detected with an overall prevalence of 77.7%, which is at the upper end of the range found in Europe [11, 26, 27]. Further sequencing of 13.1% *Bartonella* spp.-positive samples showed the presence of *B. schoenbuchensis* and *Bartonella capreoli*. *Bartonella* spp. infection can cause long-lasting intraerythrocytic bacteraemia and endotheliotropic infection but is usually not associated with disease [24, 27]*.* However, vascular pathology can develop in a small subset of individuals and in accidental hosts [24]. The high prevalence of *Bartonella* spp. in healthy roe deer could implicate roe deer are chronically bacteraemic and a natural reservoir for *Bartonella* spp. in the Netherlands. The best model for the presence of *Bartonella* spp. included only the host nutritional condition, with the presence of *Bartonella* spp. in roe deer becoming less likely as nutritional condition improved. Establishment of *Bartonella* spp. infection could be enhanced by poor condition of hosts, either directly by reduced immunocompetence or indirectly via a greater vector infestation and increased exposure to *Bartonella* spp. Further studies are needed to acquire more solid conclusions about this association.

*Babesia* spp. DNA was present in 17.4% of the roe deer, at the lower end of the range documented in Europe [10, 17, 35, 37, 38]. *Babesia* spp. are intraerythrocytic parasites, and clinical manifestations can occur in naïve animals or when latent infections flare up under stressful conditions [39, 61]. Upon sequence analysis, *B. capreoli* was identified, as well as unexpected species, such as *B. microti*. *Babesia capreoli* infection has caused death in roe deer [39, 40]. The detection of *B. microti* was not in line with expectations, as *B. microti* is primarily encountered in voles and mice, rather than in roe deer, in which *B. microti*-like pathogens have been found only once [36, 64]. High exposure of roe deer to tick bites can enable transient presence in roe deer blood of tick-associated microorganisms [65] or only their DNA. *Babesia venatorum* was not detected but may have been among the unknowns, because roe deer have been identified as one of the primary cervid reservoirs [33]. This study found a significant inverse effect of age, consistent with some studies elsewhere [17, 38]. Calves (< 1 year old) tested positive for *Babesia* spp. more frequently than older animals (≥ 1 year old). In enzootic areas, animals infected with *Babesia* spp. at a young age may benefit from passive immunity acquired from the mother and become infected without the presence of clinical symptoms [39]. After primary infection, the infection may persist and in some pre-immunized animals might be reduced to below the level of detection [38]; alternatively, it could be cleared by the immune system, leading to long-term protective immunity which prevents reinfection with this protozoal pathogen [38]. Because of the negative association of *Babesia* with age and due to the low prevalence of *Babesia* spp., *Babesia* spp. is possibly locally enzootic in the Netherlands.

DNA of spotted fever *Rickettsia* was detected in 3.3% of the roe deer. As further typing of these samples by conventional PCR failed, the rickettsial species could not be determined, and the prevalence found in this study was therefore difficult to compare with prior PCR findings where roe deer were tested for *R. helvetica* [10, 15, 48]. The life-cycle of *Rickettsia* spp. primarily depends on transovarial transmission in vectors [66]. Hence, the relatively low prevalence found in this study may suggest that roe deer might incidentally act as a sporadic source of pathogens to vectors, relevant possibly only for spatial dissemination [67].

Genetic material was detected only once for *B. burgdorferi* s.l. and not for *B. miyamotoi,* or *N. mikurensis*. The incompetence of roe deer to function as reservoirs for *B. burgdorferi* s.l. and *N. mikurensis* corresponded with earlier studies [42, 43, 45, 46, 68, 69]. Also, none of the animals were found to be positive with BTV or EHDV, implicating neither were circulating within the Dutch roe deer population in 2010. BTV antibodies from previous years when BTV was present in the Netherlands would be expected only in adult roe deer in 2010 and may have waned. Moreover, others have reported low BTV seroprevalence in roe deer [6, 49, 70], suggesting they are a less important species in the distribution of BTV in nature [70].

In multiple host vector-borne diseases, the associations between pathogen prevalence and host densities are complex, especially when vectors use different hosts in different life stages [20, 71]. In this study, there was no evidence for an association of roe deer density with the presence or absence of the detected pathogens.

Various co-infections were encountered. The most dominant combinations included *A. phagocytophilum* and *Bartonella* spp., with or without *Babesia* spp. presence. Co-infections with two or three of these pathogens (*Bartonella* spp., *A. phagocytophilum*, *Babesia* spp.) have previously been documented in roe deer [10, 11, 17, 26, 35]. Co-infection of roe deer with *A. phagocytophilum* and *Bartonella* spp. or *Babesia* spp. occurred more frequently than could be expected by chance. For *A. phagocytophilum* and *Babesia* spp., one reason may be that they are transmitted by the same vector, *I. ricinus*. In addition, *A. phagocytophilum* infection is believed to induce immunosuppressive effects, resulting in an increase in the susceptibility to simultaneous infection with vector-borne pathogens that would normally be regulated by unimpaired lymphocytes and neutrophils [72]. In younger animals this mechanism might be most prominent, because premunition has not yet been acquired [73].

## Limitations

Despite striving for random sampling of the roe deer population, operational challenges were encountered such as the dependency on hunters for sample submission and roe deer trait information, and the differential hunting seasons for does and calves compared to bucks. There was a slight but significant underrepresentation of bucks in the sample, largely due to a lower return rate of samples during the buck hunting season (59% of the sample packages assigned to GMUs in buck hunting season) compared to the doe-and-calf hunting season (69% of the sample packages assigned to GMUs in doe-and-calf hunting season). Interviews following the doe-and-calf hunting season indicated that organizational problems and suspended hunting activity are the main reasons given for the non-return of samples. Also, roe deer from GMUs with a low density (≤ 2 roe deer per 100 ha) were underrepresented. This could be a possible source of bias for the measured prevalence of pathogens and the lack of association between their presence and roe deer density. Among the roe deer trait information that the hunters supplied, age, nutritional condition and health status were not measured but estimated. Body mass, however, was significantly positively associated with male sex, increasing age, better nutritional condition, healthy status and living in areas with lower roe deer densities. This finding confirmed expectations, supporting overall correspondence in host factor information supplied by the hunters.

The differential hunting season was an unavoidable, but possible, source of bias, given that greater tick activity can normally be expected in spring and summer when the buck hunting season occurs. Therefore, as detailed previously, a seasonal effect, for example greater tick load, cannot be excluded as explanation for the positive association between male sex and *A. phagocytophilum* load. However, any bias due to a greater tick load on bucks would only have weakened the inverse association between age category and presence of *A. phagocytophilum, Babesia* spp. or co-infection. In regard to the pathogens detected, both orbiviruses were only measured during the doe-and-calf hunting season and 2 years after the last documented presence of BTV-8 in the Netherlands. The absence in roe deer positive for BTV could therefore have resulted from the disappearance of antibodies over this period of time in roe deer of > 2 years of age and absence in the younger ones.

The interpretation of the prevalence was impaired by the lack of ecotype characterisation of *A. phagocytophilum*, no identification of species involved in the possibly dual or multiple infections of *Bartonella* spp. and *Babesia* spp., and by the lack of species determination for many *Bartonella* and *Rickettsia* species. Finally, the identification of genetic material of pathogens does not necessarily identify an active infection in roe deer, but it is a strong indication that a microorganism has been present in the host.

## Conclusion

This study has given valuable information on *A. phagocytophilum*, *Bartonella* spp., *Babesia* spp., *Rickettsia* spp., *B. burgdorferi* s.l., *B. miyamotoi*, *N. mikurensis*, EHDV prevalence and BTV seroprevalence in Dutch roe deer. The high levels of infection and co-infection implicate that roe deer are of importance in the nationwide transmission of individual and multiple vector-borne pathogens in the Netherlands. Caution is warranted, however, when extrapolating prevalence of *A. phagocytophilum*, *Bartonella* spp. and *Babesia* spp. to a potential impact on public health and livestock, as further genetic characterization of the pathogens is required for a proper understanding of possible implications [73]. Age and nutritional condition were found to be the most influential host variables in regard to the presence of vector-borne pathogens in roe deer. Vector-borne pathogen presence was not associated with local roe deer density.

## Supplementary Information

**Additional file 1.** VBD Excel file.

**Additional file 2.** GMU Excel file.

**Additional file 3.** R-script.

**Additional file 4.** Table. Mean weight of sampled roe deer per age category per sex (*n* = 366).

**Additional file 5.** Sequence results.

## Data Availability

Data supporting the conclusions of this article are included within the article and its additional files. A limited amount of DNA from roe deer samples is available upon reasonable request.

## Abbreviations

AIC: Akaike information criterion
BTV: Bluetongue virus
CI: Confidence interval
C_q_: Cycle threshold
EHDV: Epizootic haemorrhagic disease virus
EDTA: Ethylenediaminetetraacetic acid
GLM: Generalized linear model
GMU: Game management unit
LR: Linear regression
MLR: Multiple linear regression
NA: Not available
OR: Odds ratio
PCR: Polymerase chain reaction
s.l.: Sensu lato
*χ*^2^: Chi-square
