# Supplementary material for: Prevalence and predictors of vector-borne pathogens in Dutch roe deer
Source: Parasit Vectors. 2022 Mar 5;15:76. doi: 10.1186/s13071-022-05195-w (PMC8898454; doi:10.1186/s13071-022-05195-w)
Supplement: Supplementary file 4 — Additional file 4. Table. Mean weight of sampled roe deer per age category per sex (n = 366). [file 13071_2022_5195_MOESM4_ESM.docx]

**Additional file 4 - Mean weight of sampled roe deer per age category per sex (n = 366)**

| **Sex** | **Age category** | **Weight (kg) ± SD (subsample size)** |
| --- | --- | --- |
| Female | Calf | 10.5 ± 1.6 (n = 93) |
|  | Yearling^a^ | 13.9 ± 2.1 (n = 45) |
|  | Adult | 15.4 ± 2.1 (n = 110) |
|  | Total female | 13.3 ± 2.9 (n = 248) |
| Male | Calf | 11.0 ± 2.1 (n = 31) |
|  | Yearling | 13.6 ± 1.8 (n = 30) |
|  | Adult | 16.7 ± 2.6 (n = 57) |
|  | Total male | 14.4 ± 3.3 (n = 118) |

^a^ Note that the doe-and-calf hunting season coincides with the timing of embryo implantation in does, and pregnancy was observed among the yearling (31 yes, 19 no, 6 NA) and adult females (116 yes, 11 no, 6 NA).
