# Supplementary material for: Prevalence and predictors of vector-borne pathogens in Dutch roe deer
Source: Parasit Vectors. 2022 Mar 5;15:76. doi: 10.1186/s13071-022-05195-w (PMC8898454; doi:10.1186/s13071-022-05195-w)
Supplement: Supplementary file 5 — Additional file 5. Sequence results. [file 13071_2022_5195_MOESM5_ESM.docx]

**Additional file 5 - Sequence results**

**BARTONELLA SCHOENBUCHENSIS (n=21)**

>B2S880 (Bartonella schoenbuchensis)

GGGGACCAGCTCATGGTGGTGCTAATGAAGCATGCTTAAAAATGCTGCAAGAAATAGGTTCTATTGAAAAAATTCCTGAGTTTATCGCACGTGCAAAAGATAAAAATGATCCTTTCCGTCTTATGGGCTTTGGCCACAGAGTTTATAAAAATTACGATCCACGTGCAAAACTTATGCAAAAAACCTGCCATGAAGTTTTAAAAGAACTAAATATTAAAGATGATCCACTTCTTGACATTGCTATGGAGCTTGAAAAAATTGCCTTAAGTGATGAATACTTTATTGAAAAAAAGCTTTATCCTAATGTTGATTTCTATTCTGGAATTACATTAAAAGCTCTAGGCTTTCCTACTGAAATGTTTAC

>B3S1343 (Bartonella schoenbuchensis)

GGACCAGCTCATGGTGGTGCTAATGAAGCATGCTTAAAAATGCTGCAAGAAATAGGTTCTATTGAAAAAAATTCCTGAGTTTATCGCACGTGCAAAAGATAAAAATGATCCTTTCCGTCTTATGGGCTTTGGCCACAGAGTTTATAAAAATTACGATCCACGTGCAAAACTTATGCAAAAAACCTGCCATGAAGTTTTAAAAGAACTAAATATTAAAGATGATCCACTTCTTGACATTGCTATGGAGCTTGAAAAAATTGCCTTAAGTGATGAATACTTTATTGAAAAAAAGCTTTATCCTAATGTTGATTTCTATTCTGGAATTACATTAAAAGCTCTAGGCTTTCCTACTGAAATGTTTACT

>B1S754 (Bartonella schoenbuchensis)

GGACCAGCTCATGGTGGTGCTAATGAAGCATGCTTAAAAATGCTGCAAGAAATAGGTTCTATTGAAAAAATTCCTGAGTTTATCGCACGTGCAAAAGATAAAAATGATCCTTTCCGTCTTATGGGCTTTGGCCACAGAGTTTATAAAAATTACGATCCACGTGCAAAACTTATGCAAAAAACCTGCCATGAAGTTTTAAAAGAACTAAATATTAAAGATGATCCACTTCTTGACATTGCTATGGAGCTTGAAAAAATTGCCTTAAGTGATGAATACTTTATTGAAAAAAAGCTTTATCCTAATGTTGATTTCTATTCTGGAATTACATTAAAAGCTCTAGGCTTTCCTACTGAAATGTTTACTGTTCTTTTTGCAT

>B1S761 (Bartonella schoenbuchensis)

GGACCAGCTCATGGTGGTGCTAATGAAGCATGCTTAAAAATGCTGCAAGAAATAGGTTCTATTGAAAAAATTCCTGAGTTTATCGCACGTGCAAAAGATAAAAATGATCCTTTCCGTCTTATGGGCTTTGGCCACAGAGTTTATAAAAATTACGATCCACGTGCAAAACTTATGCAAAAAACCTGCCATGAAGTTTTAAAAGAACTAAATATTAAAGATGATCCACTTCTTGACATTGCTATGGAGCTTGAAAAAATTGCCTTAAGTGATGAATACTTTATTGAAAAAAAGCTTTATCCTAATGTTGATTTCTATTCTGGAATTACATTAAAAGCTCTAGGCTTTCCTACTGAAATGTTTACTGTTCTTTTTGCAT

>B1S1977 (Bartonella schoenbuchensis)

GGACCAGCTCATGGTGGTGCTAATGAAGCATGCTTAAAAATGCTGCAAGAAATAGGTTCTATTGAAAAAATTCCTGAGTTTATCGCACGTGCAAAAGATAAAAATGATCCTTTCCGTCTTATGGGCTTTGGCCACAGAGTTTATAAAAATTACGATCCACGTGCAAAACTTATGCAAAAAACCTGCCATGAAGTTTTAAAAGAACTAAATATTAAAGATGATCCACTTCTTGACATTGCTATGGAGCTTGAAAAAATTGCCTTAAGTGATGAATACTTTATTGAAAAAAAGCTTTATCCTAATGTTGATTTCTATTCTGGAATTACATTAAAAGCTCTAGGCTTTCCTACTGAAATGTTTACTGTTCTTTTTGCAT

>B2S116 (Bartonella schoenbuchensis)

GGACCAGCTCATGGTGGTGCTAATGAAGCATGCTTAAAAATGCTGCAAGAAATAGGTTCTATTGAAAAAATTCCTGAGTTTATCGCACGTGCAAAAGATAAAAATGATCCTTTCCGTCTTATGGGCTTTGGCCACAGAGTTTATAAAAATTACGATCCACGTGCAAAACTTATGCAAAAAACCTGCCATGAAGTTTTAAAAGAACTAAATATTAAAGATGATCCACTTCTTGACATTGCTATGGAGCTTGAAAAAATTGCCTTAAGTGATGAATACTTTATTGAAAAAAAGCTTTATCCTAATGTTGATTTCTATTCTGGAATTACATTAAAAGCTCTAGGCTTTCCTACTGAAATGTTTACTGTTCTTTTTGCAT

>B3S1344 (Bartonella schoenbuchensis)

GGACCAGCTCATGGTGGTGCTAATGAAGCATGCTTAAAAATGCTGCAAGAAATAGGTTCTATTGAAAAAATTCCTGAGTTTATCGCACGTGCAAAAGATAAAAATGATCCTTTCCGTCTTATGGGCTTTGGCCACAGAGTTTATAAAAATTACGATCCACGTGCAAAACTTATGCAAAAAACCTGCCATGAAGTTTTAAAAGAACTAAATATTAAAGATGATCCACTTCTTGACATTGCTATGGAGCTTGAAAAAATTGCCTTAAGTGATGAATACTTTATTGAAAAAAAGCTTTATCCTAATGTTGATTTCTATTCTGGAATTACATTAAAAGCTCTAGGCTTTCCTACTGAAATGTTTACTGTTCTTTTTGCAT

>B3S774 (Bartonella schoenbuchensis)

GGACCAGCTCATGGTGGTGCTAATGAAGCATGCTTAAAAATGCTGCAAGAAATAGGTTCTATTGAAAAAATTCCTGAGGTTTATCGCACGTGCAAAAGATAAAAATGATCCTTTCCGTCTTATGGGCTTTGGCCACAGAGTTTATAAAAATTACGATCCACGTGCAAAACTTATGCAAAAAACCTGCCATGAAGTTTTAAAAGAACTAAATATTAAAGATGATCCACTTCTTGACATTGCTATGGAGCTTGAAAAAATTGCCTTAAGTGATGAATACTTTATTGAAAAAAAGCTTTATCCTAATGTTGATTTCTATTCTGGAATTACATTAAAAGCTCTAGGCTTTCCTACTGAAATGTTTACTGTTCTTTTTGCAT

>B3S773 (Bartonella schoenbuchensis)

GGGACCAGCTCATGGTGGTGCTAATGAAGCATGCTTAAAAATGCTGCAAGAAATAGGTTCTATTGAAAAAATTCCTGAGTTTATCGCACGTGCAAAAGATAAAAATGATCCTTTCCGTCTTATGGGCTTTGGCCACAGAGTTTATAAAAATTACGATCCACGTGCAAAACTTATGCAAAAAACCTGCCATGAAGTTTTAAAAGAACTAAATATTAAAGATGATCCACTTCTTGACATTGCTATGGAGCTTGAAAAAATTGCCTTAAGTGATGAATACTTTATTGAAAAAAAGCTTTATCCTAATGTTGATTTCTATTCTGGAATTACATTAAAAGCTCTAGGCTTTCCTACTGAAATGTTTACTGTTCTTTTTGCATT

>B3S779 (Bartonella schoenbuchensis)

GGGACCAGCTCATGGTGGTGCTAATGAAGCATGCTTAAAAATGCTGCAAGAAATAGGTTCTATTGAAAAAATTCCTGAGTTTATCGCACGTGCAAAAGATAAAAATGATCCTTTCCGTCTTATGGGCTTTGGCCACAGAGTTTATAAAAATTACGATCCACGTGCAAAACTTATGCAAAAAACCTGCCATGAAGTTTTAAAAGAACTAAATATTAAAGATGATCCACTTCTTGACATTGCTATGGAGCTTGAAAAAATTGCCTTAAGTGATGAATACTTTATTGAAAAAAAGCTTTATCCTAATGTTGATTTCTATTCTGGAATTACATTAAAAGCTCTAGGCTTTCCTACTGAAATGTTTACTGTTCTTTTTGCATT

>B1S1974 (Bartonella schoenbuchensis)

GGACCAGCTCATGGTGGTGCTAATGAAGCATGCTTAAAAATGCTGCAAGAAATAGGTTCTATTGAAAAAATTCCTGAGTTTATCGCACGTGCAAAAGATAAAAATGATCCTTTCCGTCTTATGGGCTTTGGCCACAGAGTTTATAAAAATTACGATCCACGTGCAAAACTTATGCAAAAAACCTGCCATGAAGTTTTAAAAGAACTAAATATTAAAGATGATCCACTTCTTGACATTGCTATGGAGCTTGAAAAAATTGCCTTAAGTGATGAATACTTTATTGAAAAAAAGCTTTATCCTAATGTTGATTTCTATTCTGGAATTACATTAAAAGCTCTAGGCTTTCCTACTGAAATGTTTACTGTTCTTTTTGCATT

>B1S1975 (Bartonella schoenbuchensis)

GGACCAGCTCATGGTGGTGCTAATGAAGCATGCTTAAAAATGCTGCAAGAAATAGGTTCTATTGAAAAAATTCCTGAGTTTATCGCACGTGCAAAAGATAAAAATGATCCTTTCCGTCTTATGGGCTTTGGCCACAGAGTTTATAAAAATTACGATCCACGTGCAAAACTTATGCAAAAAACCTGCCATGAAGTTTTAAAAGAACTAAATATTAAAGATGATCCACTTCTTGACATTGCTATGGAGCTTGAAAAAATTGCCTTAAGTGATGAATACTTTATTGAAAAAAAGCTTTATCCTAATGTTGATTTCTATTCTGGAATTACATTAAAAGCTCTAGGCTTTCCTACTGAAATGTTTACTGTTCTTTTTGCATT

>B3S778 (Bartonella schoenbuchensis)

GGACCAGCTCATGGTGGTGCTAATGAAGCATGCTTAAAAATGCTGCAAGAAATAGGTTCTATTGAAAAAATTCCTGAGTTTATCGCACGTGCAAAAGATAAAAATGATCCTTTCCGTCTTATGGGCTTTGGCCACAGAGTTTATAAAAATTACGATCCACGTGCAAAACTTATGCAAAAAACCTGCCATGAAGTTTTAAAAGAACTAAATATTAAAGATGATCCACTTCTTGACATTGCTATGGAGCTTGAAAAAATTGCCTTAAGTGATGAATACTTTATTGAAAAAAAGCTTTATCCTAATGTTGATTTCTATTCTGGAATTACATTAAAAGCTCTAGGCTTTCCTACTGAAATGTTTACTGTTCTTTTTGCATT

>B3S1345 (Bartonella schoenbuchensis)

GGACCAGCTCATGGTGGTGCTAATGAAGCATGCTTAAAAATGCTGCAAGAAATAGGTTCTATTGAAAAAATTCCTGAGTTTATCGCACGTGCAAAAGATAAAAATGATCCTTTCCGTCTTATGGGCTTTGGCCACAGAGTTTATAAAAATTACGATCCACGTGCAAAACTTATGCAAAAAACCTGCCATGAAGTTTTAAAAGAACTAAATATTAAAGATGATCCACTTCTTGACATTGCTATGGAGCTTGAAAAAATTGCCTTAAGTGATGAATACTTTATTGAAAAAAAGCTTTATCCTAATGTTGATTTCTATTCTGGAATTACATTAAAAGCTCTAGGCTTTCCTACTGAAATGTTTACTGTTCTTTTTGCATT

>B1S1972 (Bartonella schoenbuchensis)

GGGGACCAGCTCATGGTGGTGCTAATGAAGCATGCTTAAAAATGCTGCAAGAAATAGGTTCTATTGAAAAAATTCCTGAGTTTATCGCACGTGCAAAAGATAAAAATGATCCTTTCCGTCTTATGGGCTTTGGCCACAGAGTTTATAAAAATTACGATCCACGTGCAAAACTTATGCAAAAAACCTGCCATGAAGTTTTAAAAGAACTAAATATTAAAGATGATCCACTTCTTGACATTGCTATGGAGCTTGAAAAAATTGCCTTAAGTGATGAATACTTTATTGAAAAAAAGCTTTATCCTAATGTTGATTTCTATTCTGGAATTACATTAAAAGCTCTAGGCTTTCCTACTGAAATGTTTACTGTTCTTTTTGCATT

>B3S775 (Bartonella schoenbuchensis)

GGGGACCAGCTCATGGTGGTGCTAATGAAGCATGCTTAAAAATGCTGCAAGAAATAGGTTCTATTGAAAAAATTCCTGAGTTTATCGCACGTGCAAAAGATAAAAATGATCCTTTCCGTCTTATGGGCTTTGGCCACAGAGTTTATAAAAATTACGATCCACGTGCAAAACTTATGCAAAAAACCTGCCATGAAGTTTTAAAAGAACTAAATATTAAAGATGATCCACTTCTTGACATTGCTATGGAGCTTGAAAAAATTGCCTTAAGTGATGAATACTTTATTGAAAAAAAGCTTTATCCTAATGTTGATTTCTATTCTGGAATTACATTAAAAGCTCTAGGCTTTCCTACTGAAATGTTTACTGTTCTTTTTGCATT

>B1S1980 (Bartonella schoenbuchensis)

GGGACCAGCTCATGGTGGTGCTAATGAAGCATGCTTAAAAATGCTGCAAGAAATAGGTTCTATTGAAAAAATTCCTGARTTTATCGCACGTGCAAAAGATAAAAATGATCCTTTCCGTCTTATGGGCTTTGGCCACAGAGTTTATAAAAATTAYGATCCACGTGCAAAACTTATGCAAAAAACCTGCCATGAAGTTTTAAAAGAACTMAATATTAAAGATGATCCACTTCTTGACATTGCTATGGARCTTGAAAAAATTGCYTTRAGTGATGAATACTTTATTGAAAAAAAGCTTTATCCTAATGTTGATTTCTATTCTGGAATTACATTAAAAGCTCTAGGCTTTCCTACYGAAATGTTTACTGTTCTTTTTGCATT

>B3S1347 (Bartonella schoenbuchensis)

GAAAAAAATTCCTGAGTTTATCGCACGTGCAAAAGATAAAAATGATCCTTTCCGTCTTATGGGCTTTGGCCACAGAGTTTATAAAAATTACGATCCACGTGCAAAACTTATGCAAAAAACCTGCCATGAAGTTTTAAAAGAACTAAATATTAAAGATGATCCACTTCTTGACATTGCTATGGAGCTTGAAAAAATTGCCTTAAGTGATGAATACTTTATTGAAAAAAAGCTTTATCCTARTGTTGATTTCTATTCTGGAATTACATTAAAAGCTCTAGGCTTTCCTACTGAAATGTTTAC

>B3S1346 (Bartonella schoenbuchensis)

AAAAATGCTGCAAGAAATAGGTTTCTWTKRAAAAATTCCTGAGTTTATCGCACGTGCAAAAGATAAAAATGATCCTTTCCGTCTTATGGGCTTTGGCCACAGAGTTTATAAAAATTACGATCCACGTGCAAAACTTATGCAAAAAACCTGCCATGAAGTTTTAAAAGAACTAAATATTAAAGATGATCCACTTCTTGACATTGCTATGGAGCTTGAAAAAATTGCCTTAAGTGATGAATACTTTATTGAAAAAAAGCTTTATCCTAATGTTGATTTCTATTCTGGAATTACATTAAAAGCTCTAGGCTTTCCTACTGAAATGTTT

> B3 S4 55

ggaccagctcatggtggtgctaatgaagcatgcttaaaaatgctgcaagaaataggttctattgaaaaaattcctgagtttatcgcacgtgcaaaagataaaaatgatcctttccgtcttatgggctttggccacagagtttataaaaattacgatccacgtgcaaaacttatgcaaaaaacctgccatgaagttttaaaagaactaaatattaaagatgatccacttcttgacattgctatggagcttgaaaaaattgccttaagtgatgaatactttattgaaaaaaagctttatcctaatgttgatttctattctggaattacattaaaagctctaggctttcctactgaaatgtttactgttctttttgcatt

>B3 S4 56

ggaccagctcatggtggtgctaatgaagcatgcttaaaaatgctgcaagaaataggttctattgaaaaaattcctgagtttatcgcacgtgcaaaagataaaaatgatcctttccgtcttatgggctttggccacagagtttataaaaattacgatccacgtgcaaaacttatgcaaaaaacctgccatgaagttttaaaagaactaaatattaaagatgatccacttcttgacattgctatggagcttgaaaaaattgccttaagtgatgaatactttattgaaaaaaagctttatcctaatgttgatttctattctggaattacattaaaagctctaggctttcctactgaaatgtttactgttctttttgcatt

**BARTONELLA CAPREOLI (N=5)**

>B1S757 (Bartonella capreoli)

GGACCAGCTCATGGTGGTGCTAATGAAGCATGCTTAAAAATGCTGCAAGAAATAGGCTCTATTGAAAAAATTCCTGAATTTATCGCACGTGCAAAAGATAAAAATGATCCTTTCCGTCTTATGGGCTTTGGCCACAGAGTTTATAAAAATTATGATCCACGTGCAAAACTTATGCAAAAAACCTGCCATGAAGTTTTAAAAGAACTCAATATTAAAGATGATCCACTTCTTGACATTGCTATGGAACTTGAAAAAATTGCTCTGAGTGATGAATACTTTATTGAAAAAAAGCTCTATCCTAATGTTGATTTCTATTCTGGAATTACATTAAAAGCTCTAGGCTTTCCTACCGAAATGTTTACTGTTCTTTTTGCAT

>B2S82 (Bartonella capreoli)

GGGACCAGCTCATGGTGGTGCTAATGAAGCATGCTTAAAAATGCTGCAAGAAATAGGCTCTATTGAAAAAATTCCTGAATTTATCGCACGTGCAAAAGATAAAAATGATCCTTTCCGTCTTATGGGCTTTGGCCACAGAGTTTATAAAAATTATGATCCACGTGCAAAACTTATGCAAAAAACCTGCCATGAAGTTTTAAAAGAACTCAATATTAAAGATGATCCACTTCTTGACATTGCTATGGAACTTGAAAAAATTGCTCTGAGTGATGAATACTTTATTGAAAAAAAGCTCTATCCTAATGTTGATTTCTATTCTGGAATTACATTAAAAGCTCTAGGCTTTCCTACCGAAATGTTTACTGTTCTTTTTGCAT

>B3S780 (Bartonella capreoli)

GGACCAGCTCATGGTGGTGCTAATGAAGCATGCTTAAAAATGCTGCAAGAAATAGGYTCTATTGAAAAAATTCCTGAATTTATCGCACGTGCAAAAGATAAAAATGATCCTTTCCGTCTTATGGGCTTTGGCCACAGAGTTTATAAAAATTATGATCCACGTGCAAAACTTATGCAAAAAACCTGCCATGAAGTTTTAAAAGAACTCAATATTAAAGATGATCCACTTCTTGACATTGCTATGGAACTTGAAAAAATTGCTCTGAGTGATGAATACTTTATTGAAAAAAAGCTCTATCCTAATGTTGATTTCTATTCTGGAATTACATTAAAAGCTCTAGGCTTTCCTACCGAAATGTTTACTGTTCTTTTTGCAT

>B3S777 (Bartonella capreoli)

GGACCAGCTCATGGTGGTGCTAATGAAGCATGCTTAAAAATGCTGCAAGAAATAGGYTCTATTGAAAAAATTCCTGANTTTATCGCACGTGCAAAAGATAAAAATGATCCTTTCCGTCTTATGGGCTTTGGCCACAGAGTTTATAAAAATTAYGATCCACGTGCAAAACTTATGCAAAAAACCTGCCATGAAGTTTTAAAAGAACTMAATATTAAAGATGATCCACTTCTTGACATTGCTATGGAACTTGAAAAAATTGCTYTRAGTGATGAATACTTTATTGAAAAAAAGCTYTATCCTAATGTTGATTTCTATTCTGGAATTACATTAAAAGCTCTAGGCTTTCCTACYGAAATGTTTACTGTTCTTTTTGCAT

>B2S81 (Bartonella capreoli)

GGACCAGCTCATGGTGGTGCTAATGAAGCATGCTTAAAAATGCTGCAAGAAATAGGYTCTATTGAAAAAATTCCTGARTTTATCGCACGTGCAAAAGATAAAAATGATCCTTTCCGTCTTATGGGCTTTGGCCACAGAGTTTATAAAAATTAYGATCCACGTGCAAAACTTATGCAAAAAACCTGCCATGAAGTTTTAAAAGAACTMAATATTAAAGATGATCCACTTCTTGACATTGCTATGGARCTTGAAAAAATTGCYYTRAGTGATGAATACTTTATTGAAAAAAAGCTYTATCCTAATGTTGATTTCTATTCTGGAATTACATTAAAAGCTCTAGGCTTTCCT

**BABESIA MICROTI (n=3)**

>B2S335 (Babesia microti)

aaataacaatacagggcttaaagtcctgtaattggaatgatgggaatctaaacccttcccagagtatcaattggagggcaagtctggtgccagcagccgcggtaattccagctccaatagcgtatattaaagttgttgcagttaagaagctcgtagttgaatttctgccttgtcattaatctcgcttccgagcgtttttttattgacttggcatcttctggatttggtgccttcgggtactattttccaggatttactttgagaaaactagagtgtttcaaacaggcattcgccttgaatactacagcatggaataatgaagtagtactttggttctattttgttggttattgagccagagtaatggttaataggagcagttgggggcattcgtatt

>B2S336 (Babesia microti)

Aaataacaatacagggyttaaagtcctgtaattggaatgatgggaatctaaacccttcccagagtatcaattggagggcaagtctggtgccagcagccgcggtaattccagctccaatagcgtatattaaagttgttgcagttaagaagctcgtagttgaatttctgccttgtcattaatctcgcttccgagcgtttttttattgacttggcatcttctggatttggtgccttcgggtactattttccaggatttactttgagaaaactagagtgtttcaaacaggcattcgccttgaatactacagcatggaataatgaagtagtactttggttctattttgttggttattgagccaraktaatggttaataggagcagttgggggcattcgtatt

>B2S337 (Babesia microti)

aaataacaatacagggcttaaagtcctgtaattggaatgatgggaatctaaacccttcccagagtatcaattggagggcaagtctggtgccagcagccgcggtaattccagctccaatagcgtatattaaagttgttgcagttaagaagctcgtagttgaatttctgccttgtcattaatctcgcttccgagcgtttttttattgacttggcatcttctggatttggtgccttcgggtactattttccaggatttactttgagaaaactagagtgtttcaaacaggcattcgccttgaatactacagcatggaataatgaagtagtactttggttctattttgttggttattgagccagagtaatggttaataggagcagttgggggcattcgtatt

**BABESIA CAPREOLI (N=27)**

>B1 S1 7 (Babesia capreoli)

AAATAACAATACAGGGCAATTGTCTTGTAATTGGAATGATGGTGACCTAAACCCTCACCAGAGTAACAATTGGAGGGCAAGTCTGGTGCCAGCAGCCGCGGTAATTCCAGCTCCAATAGCGTATATTAAACTTGTTGCAGTTAAAAAGCTCGTAGTTGAATTTTTGCGTGGTGTTAATATTGACTGATGTCGAGATTGCACTTCGCTTTTGGGATTTTTCCCTTTTTACTTTGAGAAAATTAGAGTGTTTCAAGCAGACTTTTGTCTTGAATACTTCAGCATGGAATAATAGAGTAGGACTTTGGTTCTATTTTGTTGGTTTGTGAACCTTAGTAATGGTTAATAGGAACGGTTGGGGGCATTCGTATT

>B1 S20 1 (Babesia capreoli)

AAATAACAATACAGGGCAATTGTCTTGTAATTGGAATGATGGTGACCTAAACCCTCACCAGAGTAACAATTGGAGGGCAAGTCTGGTGCCAGCAGCCGCGGTAATTCCAGCTCCAATAGCGTATATTAAACTTGTTGCAGTTAAAAAGCTCGTAGTTGAATTTTTGCGTGGTGTTAATATTGACTGATGTCGAGATTGCACTTCGCTTTTGGGATTTTTCCCTTTTTACTTTGAGAAAATTAGAGTGTTTCAAGCAGACTTTTGTCTTGAATACTTCAGCATGGAATAATAGAGTAGGACTTTGGTTCTATTTTGTTGGTTTGTGAACCTTAGTAATGGTTAATAGGAACGGTTGGGGGCATTCGTATT

>B1 S3 22 (Babesia capreoli)

AAATAACAATACAGGGCAATTGTCTTGTAATTGGAATGATGGTGACCTAAACCCTCACCAGAGTAACAATTGGAGGGCAAGTCTGGTGCCAGCAGCCGCGGTAATTCCAGCTCCAATAGCGTATATTAAACTTGTTGCAGTTAAAAAGCTCGTAGTTGAATTTTTGCGTGGTGTTAATATTGACTGATGTCGAGATTGCACTTCGCTTTTGGGATTTTTCCCTTTTTACTTTGAGAAAATTAGAGTGTTTCAAGCAGACTTTTGTCTTGAATACTTCAGCATGGAATAATAGAGTAGGACTTTGGTTCTATTTTGTTGGTTTGTGAACCTTAGTAATGGTTAATAGGAACGGTTGGGGGCATTCGTATT

>B2 S17 72 (Babesia capreoli)

AAATAACAATACAGGGCAATTGTCTTGTAATTGGAATGATGGTGACCTAAACCCTCACCAGAGTAACAATTGGAGGGCAAGTCTGGTGCCAGCAGCCGCGGTAATTCCAGCTCCAATAGCGTATATTAAACTTGTTGCAGTTAAAAAGCTCGTAGTTGAATTTTTGCGTGGTGTTAATATTGACTGATGTCGAGATTGCACTTCGCTTTTGGGATTTTTCCCTTTTTACTTTGAGAAAATTAGAGTGTTTCAAGCAGACTTTTGTCTTGAATACTTCAGCATGGAATAATAGAGTAGGACTTTGGTTCTATTTTGTTGGTTTGTGAACCTTAGTAATGGTTAATAGGAACGGTTGGGGGCATTCGTATT

>B2 S20 13 (Babesia capreoli)

AAATAACAATACAGGGCAATTGTCTTGTAATTGGAATGATGGTGACCTAAACCCTCACCAGAGTAACAATTGGAGGGCAAGTCTGGTGCCAGCAGCCGCGGTAATTCCAGCTCCAATAGCGTATATTAAACTTGTTGCAGTTAAAAAGCTCGTAGTTGAATTTTTGCGTGGTGTTAATATTGACTGATGTCGAGATTGCACTTCGCTTTTGGGATTTTTCCCTTTTTACTTTGAGAAAATTAGAGTGTTTCAAGCAGACTTTTGTCTTGAATACTTCAGCATGGAATAATAGAGTAGGACTTTGGTTCTATTTTGTTGGTTTGTGAACCTTAGTAATGGTTAATAGGAACGGTTGGGGGCATTCGTATT

>B2 S21 16 (Babesia capreoli)

AAATAACAATACAGGGCAATTGTCTTGTAATTGGAATGATGGTGACCTAAACCCTCACCAGAGTAACAATTGGAGGGCAAGTCTGGTGCCAGCAGCCGCGGTAATTCCAGCTCCAATAGCGTATATTAAACTTGTTGCAGTTAAAAAGCTCGTAGTTGAATTTTTGCGTGGTGTTAATATTGACTGATGTCGAGATTGCACTTCGCTTTTGGGATTTTTCCCTTTTTACTTTGAGAAAATTAGAGTGTTTCAAGCAGACTTTTGTCTTGAATACTTCAGCATGGAATAATAGAGTAGGACTTTGGTTCTATTTTGTTGGTTTGTGAACCTTAGTAATGGTTAATAGGAACGGTTGGGGGCATTCGTATT

>B2 S4 51 (Babesia capreoli)

AAATAACAATACAGGGCAATTGTCTTGTAATTGGAATGATGGTGACCTAAACCCTCACCAGAGTAACAATTGGAGGGCAAGTCTGGTGCCAGCAGCCGCGGTAATTCCAGCTCCAATAGCGTATATTAAACTTGTTGCAGTTAAAAAGCTCGTAGTTGAATTTTTGCGTGGTGTTAATATTGACTGATGTCGAGATTGCACTTCGCTTTTGGGATTTTTCCCTTTTTACTTTGAGAAAATTAGAGTGTTTCAAGCAGACTTTTGTCTTGAATACTTCAGCATGGAATAATAGAGTAGGACTTTGGTTCTATTTTGTTGGTTTGTGAACCTTAGTAATGGTTAATAGGAACGGTTGGGGGCATTCGTATT

>B3 S5 58 (Babesia capreoli)

AAATAACAATACAGGGCAATTGTCTTGTAATTGGAATGATGGTGACCTAAACCCTCACCAGAGTAACAATTGGAGGGCAAGTCTGGTGCCAGCAGCCGCGGTAATTCCAGCTCCAATAGCGTATATTAAACTTGTTGCAGTTAAAAAGCTCGTAGTTGAATTTTTGCGTGGTGTTAATATTGACTGATGTCGAGATTGCACTTCGCTTTTGGGATTTTTCCCTTTTTACTTTGAGAAAATTAGAGTGTTTCAAGCAGACTTTTGTCTTGAATACTTCAGCATGGAATAATAGAGTAGGACTTTGGTTCTATTTTGTTGGTTTGTGAACCTTAGTAATGGTTAATAGGAACGGTTGGGGGCATTCGTATT

>B1 S17 63 (Babesia capreoli)

AAATAACAATACAGGGCAATTGTCTTGTAATTGGAATGATGGTGACCTAAACCCTCACCAGAGTAACAATTGGAGGGCAAGTCTGGTGCCAGCAGCCGCGGTAATTCCAGCTCCAATAGCGTATATTAAACTTGTTGCAGTTAAAAAGCTCGTAGTTGAATTTTTGCGTGGTGTTAATATTGACTGATGTCGAGATTGCACTTCGCTTTTGGGATTTTTCCCTTTTTACTTTGAGAAAATTAGAGTGTTTCAAGCAGACTTTTGTCTTGAATACTTCAGCATGGAATAATAGAGTAGGACTTTGGTTCTATTTTGTTGGTTTGTGAACCTTAGTAATGGTTAATAGGAACGGTTGGGG

>B2 S6 62 (Babesia capreoli)

AAATAACAATACAGGGCAATTGTCTTGTAATTGGAATGATGGTGACCTAAACCCTCACCAGAGTAACAATTGGAGGGCAAGTCTGGTGCCAGCAGCCGCGGTAATTCCAGCTCCAATAGCGTATATTAAACTTGTTGCAGTTAAAAAGCTCGTAGTTGAATTTTTGCGTGGTGTTAATATTGACTGATGTCGAGATTGCACTTCGCTTTTGGGATTTTTCCCTTTTTACTTTGAGAAAATTAGAGTGTTTCAAGCAGACTTTTGTCTTGAATACTTCAGCATGGAATAATAGAGTAGGACTTTGGTTCTATTTTGTTGGTTTGTGAACCTTAGTAATGGTTAATAGGAACGGTTGGGG

>B2 S18 80 (Babesia capreoli)

AAATAACAATACAGGGCAATTGTCTTGTAATTGGAATGATGGTGACCTAAACCCTCACCAGAGTAACAATTGGAGGGCAAGTCTGGTGCCAGCAGCCGCGGTAATTCCAGCTCCAATAGCGTATATTAAACTTGTTGCAGTTAAAAAGCTCGTAGTTGAATTTTTGCGTGGTGTTAATATTGACTGATGTCGAGATTGCACTTCGCTTTTGGGATTTTTCCCTTTTTACTTTGAGAAAATTAGAGTGTTTCAAGCAGACTTTTGTCTTGAATACTTCAGCATGGAATAATAGAGTAGGACTTTGGTTCTATTTTGTTGGTTTGTGAACCTTAGTAATGGTTAATAGGAACGGTTGGG

>B2 S5 52 (Babesia capreoli)

AAATAACAATACAGGGCAATTGTCTTGTAATTGGAATGATGGTGACCTAAACCCTCACCAGAGTAACAATTGGAGGGCAAGTCTGGTGCCAGCAGCCGCGGTAATTCCAGCTCCAATAGCGTATATTAAACTTGTTGCAGTTAAAAAGCTCGTAGTTGAATTTTTGCGTGGTGTTAATATTGACTGATGTCGAGATTGCACTTCGCTTTTGGGATTTTTCCCTTTTTACTTTGAGAAAATTAGAGTGTTTCAAGCAGACTTTTGTCTTGAATACTTCAGCATGGAATAATAGAGTAGGACTTTGGTTCTATTTTGTTGGTTTGTGAACCTTARTAATGGTTAATAGGAACGGTTGGGG

>B1 S18 67 (Babesia capreoli)

AAATAACAATACAGGGCAATTGTCTTGTAATTGGAATGATGGTGACCTAAACCCTCACCAGAGTAACAATTGGAGGGCAAGTCTGGTGCCAGCAGCCGCGGTAATTCCAGCTCCAATAGCGTATATTAAACTTGTTGCAGTTAAAAAGCTCGTAGTTGAATTTTTGCGTGGTGTTAATATTGACTGATGTCGAGATTGCACTTCGCTTTTGGGATTTTTCCCTTTTTACTTTGAGAAAATTAGAGTGTTTCAAGCAGACTTTTGTCTTGAATACTTCAGCATGGAATAATAGAGTAGGACTTTGGTTCTATTTTGTTGGTTTGTGAACCTTAKTAATGGTTAATAGGAACGGTTGGG

>B2 S17 76 (Babesia capreoli)

AAATAACAATACAGGGCAATTGTCTTGTAATTGGAATGATGGTGACCTAAACCCTCACCAGAGTAACAATTGGAGGGCAAGTCTGGTGCCAGCAGCCGCGGTAATTCCAGCTCCAATAGCGTATATTAAACTTGTTGCAGTTAAAAAGCTCGTAGTTGAATTTTTGCGTGGTGTTAATATTGACTGATGTCGAGATTGCACTTCGCTTTTGGGATTTTTCCCTTTTTACTTTGAGAAAATTAGAGTGTTTCAAGCAGACTTTTGTCTTGAATACTTCAGCATGGAATAATAGAGTAGGACTTTGGTTCTATTTTGTTGGTTTGTGAACCTTAKTAATGGTTAATAGGAACGGTTGG

>B2 S14 46 (Babesia capreoli)

AAATAACAATACAGGGCAATTGTCTTGTAATTGGAATGATGGTGACCTAAACCCTCACCAGAGTAACAATTGGAGGGCAAGTCTGGTGCCAGCAGCCGCGGTAATTCCAGCTCCAATAGCGTATATTAAACTTGTTGCAGTTAAAAAGCTCGTAGTTGAATTTTTGCGTGGTGTTAATATTGACTGATGTCGAGATTGCACTTCGCTTTTGGGATTTTTCCCTTTTTACTTTGAGAAAATTAGAGTGTTTCAAGCAGACTTTTGTCTTGAATACTTCAGCATGGAATAATARAGTAGGACTTTGGTTCTATTTTGTTGGTTTGTGAACCTTAKTAATGGTTAATAGGAACGGTTGGGGGCATTCGTATT

>B3 S10 18 (Babesia capreoli)

AAATAACAATACAGGGCAATTGTCTTGTAATTGGAATGATGGTGACCTAAACCCTCACCAGAGTAACAATTGGAGGGCAAGTCTGGTGCCAGCAGCCGCGGTAATTCCAGCTCCAATAGCGTATATTAAACTTGTTGCAGTTAAAAAGCTCGTAGTTGAATTTTTGCGTGGTGTTAATATTGACTGATGTCGAGATTGCACTTCGCTTTTGGGATTTTTCCCTTTTTACTTTGAGAAAATTAGAGTGTTTCAAGCARACTTTTGTCTTGAATACTTCAGCATGGAATAATARAGTAGGACTTTGGTTCTATTTTGTTGGTTTGTGAACCTTAGTAATGGTTAATAGGAACGGTTGGGGGCATTCGTATT

>B2 S17 73 (Babesia capreoli)

AAATAWCAATACAGGGCAATTGTCTTGTAATTGGAATGATGGTGACCTAAACCCTCACCAGAGTAACAATTGGAGGGCAAGTCTGGTGCCAGCAGCCGCGGTAATTCCAGCTCCAATAGCGTATATTAAACTTGTTGCAGTTAAAAAGCTCGTAGTTGAATTTTTGCGTGGTGTTAATATTGACTGATGTCGAGATTGCACTTCGCTTTTGGGATTTTTCCCTTTTTACTTTGAGAAAATTAGAGTGTTTCAAGCAGACTTTTGTCTTGAATACTTCAKCATGGAATAATAGAGTAGGACTTTGGTTCTATTTTGTTGGTTTGTGAACCTTARTAATGGTTAATAGGAACGGTTGGGG

>B1 S15 46 (Babesia capreoli)

AAATAACAATACAGGGCAATTGTCTTGTAATTGGAATGATGGTGACCTAAACCCTCACCAGAGTAACAATTGGAGGGCAAGTCTGGTGCCAGCAGCCGCGGTAATTCCAGCTCCAATAGCGTATATTAAACTTGTTGCAGTTAAAAAGCTCGTAGTTGAATTTTTGCGTGGTGTTAATATTGACTGATGTCGAGATTGCACTTCGCTTTTGGGATTTTTCCCTTTTTACTTTGAGAAAATTAGAGTGTTTCAAGCAGACTTTTGTCTTGAATACTTCAGCATGGAATAATAGAGTAGGACTTTGGTTCTATTTTGTTGGTTTGTGAACCTTAKTAATGGTTAATAMGAACGGTTGGGGGCATTCGYATT

>B2 S13 45 (Babesia capreoli)

AAATAACAATACAGGGCAATTGTCTTRTAATTRRAATGATGGTGACCTAAACCCTCACCAGAGTAACAATTGGAGGGCAAGTCTGGTGCCAGCAGCCGCGGTAATTCCAGCTCCAATAGCGTATATTAAACTTGTTGCAGTTAAAAAGCTCGTAGTTGAATTTTTGCGTGGTGTTAATATTGACTGATGTCGAGATTGCACTTCGCTTTTGGGATTTTTCCCTTTTTACTTTGAGAAAATTAGAGTGTTTCAAGCAGACTTTTGTCTTGAATACTTCAGCATGGAATAATAGAGTAGGACTTTGGTTCTATTTTGTTGGTTTGTGAACCTTARTAATGGTTAATAGGAACGGTTGGGGGCATTCGTATT

>B2 S13 41 (Babesia capreoli)

AAATAACAATACAGGGCAATTGTNTNNTRRNNRRAATGATGGTGACCTAAACCCTCACCAGAGTAACAATTGGAGGGCAAGTCTGGTGCCAGCAGCCGCGGTAATTCCAGCTCCAATAGCGTATATTAAACTTGTTGCAGTTAAAAAGCTCGTAGTTGAATTTTTGCGTGGTGTTAATATTGACTGATGTCGAGATTGCACTTCGCTTTTGGGATTTTTCCCTTTTTACTTTGAGAAAATTAGAGTGTTTCAAGCAGACTTTTGTCTTGAATACTTCAGCATGGAATAATAGAGTAGGACTTTGGTTCTATTTTGTTGGTTTGTGAACCTTAGTAATGGTTAATAGGAACGGTTGGGG

>B1 S13 22 (Babesia capreoli)

AAATAACAATACAGGGCAATTGTCTTGTAATTGGAATGATGGTGACCTAAACCCTCACCAGAGTAACAATTGGAGGGCAAGTCTGGTGCCAGCAGCCGCGGTAATTCCAGCTCCAATAGCGTATATTAAACTTGTTGCAGTTAAAAAGCTCGTAGTTGAATTTTTGCGTGGTGTTAATATTGACTGATGTCGAGATTGCACTTCGCTTTTGGGATTTTTCCCTTTTTACTTTGAGAAAATTAGAGTGTTTCAAGCAGACTTTTGTCTTGAATACTTCAGCATGGAATAATAGAGTAGGACTTTGGTTCTATTTTGTTGGTTTGTGAACCTTAMTAATGGTTAATASGAAMGGTTGGG

>B2 S19 Fons 7 (Babesia capreoli)

AAATAACAATACAGGGCAATTGTCTTGTAATTGGAATGATGGTGACCTAAACCCTCACCAGAGTAACAATTGGAGGGCAAGTCTGGTGCCAGCAGCCGCGGTAATTCCAGCTCCAATAGCGTATATTAAACTTGTTGCAGTTAAAAAGCTCGTAGTTGAATTTTTGCGTGGTGTTAATATTGACTGATGTCGAGATTGCACTTCGCTTTTGGGATTTTTCCCTTTTTACTTTGAGAAAATTAGAGTGTTTCAAGCAGACTTTTGTCTTGAATACTTCAGCATGGAATAATAGAGTAGGACTTTGGYTCTATTTTGTTGGTTTGTGAACCTTAKTAATGGTTAATARGAACGGTTGGGGRCATTCKTATY

>B3 S10 20 (Babesia capreoli)

AAATAACAATACAGGGCAATTGTYTWGTAATTGGAATGATGGTGACCTAAACCCTCACCAGRGTAACAATTGGAGGGCAAGTCTGGTGCCAGCAGCCGCGGTAATTCCAGCTCCAATAGCGTATATTAAACTTGTTGCAGTTAAAAAGCTCGTAGTTGAATTTTTGCGTGGTGTTAATATTGACTGATGTCGAGATTGCACTTCGCTTTTGGGATTTTTCCCTTTTTACTTTGAGAAAATTAGAGTGTTTCAAGCAGACTTTTGTCTTGAATACTTCWGCATGGAATAATAGAGTARGACTTTGGTTCTATTTTGTTGGTTTGTGAACCTTAKYAATGGTTAATAGGAACGGTTGGGGGCATTCKTATT

>B2 S18 Fons 3 (Babesia capreoli)

AAATAACAATACAGGGCAATTGTCTTGTAATTGGAATGATGGTGACCTAAACCCTCACCAGAGTAACAATTGGAGGGCAAGTCTGGTGCCAGCAGCCGCGGTAATTCCAGCTCCAATAGCGTATATTAAACTTGTTGCAGTTAAAAAGCTCGTAGTTGAATTTTTGCGTGGTGTTAATATTGACTGATGTCGAGATTGCACTTCGCTTTTGGGATTTTTCCCTTTTTACTTTGAGAAAATTAGAGTGTTTCAAGCAGACTTTTGTCTTGAATACTTSWYCATGKAATAATAGAGWARGACTTTGGTTCTATTTTGTTGGTTTG

>B1 S19 77 (Babesia capreoli)

AAATAACAATACAGGGCAATTGTCTTGTAATTGGAATGATGGTGACCTAAACCCTCACCAGAGTAACAATTGGAGGGCAAGTCTGGTGCCAGCAGCCGCGGTAATTCCAGCTCCAATAGCGTATATTAAACTTGTTGCAGTTAAAAAGCTCGTAGTTGAATTTTTGCGTGGTGTTAATATTGACTGATGTCGAGATTGCACTTCGCTTTTGGGATTTTTCCCTTTTTACTTTGAGAAAATTAGAGTGTTTCAAGCAGACTTTTGTCTTGAATACTTCAGCATGGAATAATAGAGTAGGACTTTGGTTCTATTTTGTTGGTTTGTGAACCTTARTAATGGTTAATAGGAACGGTTGGGG

>B1 S19 78 (Babesia capreoli)

aaataacaatacagggcaattgtcttgtaattggaatgatggtgacctaaaccctcaccagagtaacaattggagggcaagtctggtgccagcagccgcggtaattccagctccaatagcgtatattaaacttgttgcagttaaaaagctcgtagttgaatttttgcgtggtgttaatattgactgatgtcgagattgcacttcgcttttgggatttttccctttttactttgagaaaattagagtgtttcaagcagacttttgtcttgaatacttcagcatggaataatagagtaggactttggttctattttgttggtttgtgaaccttagtaatggttaataggaacggttgggggcattcgtatt

>B2 S2 26 (Babesia capreoli)

aaataacaatacagggcaattgtcttgtaattggaatgatggtgacctaaaccctcaccagagtaacaattggagggcaagtctggtgccagcagccgcggtaattccagctccaatagcgtatattaaacttgttgcagttaaaaagctcgtagttgaatttttgcgtggtgttaatattgactgatgtcgagattgcacttcgcttttgggatttttccctttttactttgagaaaattagagtgtttcaagcagacttttgtcttgaatacttcagcatggaataatagagtaggactttggttctattttgttggtttgtgaaccttaktaatggttaataggaacggttggg
